# The importin‐alpha superfamily engages in ethylene signaling by shuttling ETHYLENE INSENSITIVE 2 from the endoplasmic reticulum to the nucleus

**DOI:** 10.1111/febs.70285

**Published:** 2025-10-13

**Authors:** Fabian Wynen, Jan Eric Maika, Raphael Josef Eberle, Nina Jahnke, Marcel Wiermer, Laura Hartmann, Rüdiger Simon, Georg Groth

**Affiliations:** ^1^ Institute of Biochemical Plant Physiology Heinrich‐Heine‐Universität Düsseldorf Germany; ^2^ Institute of Development Genetics Heinrich‐Heine‐Universität Düsseldorf Germany; ^3^ Institute for Organic Chemistry and Macromolecular Chemistry Heinrich‐Heine‐Universität Düsseldorf Germany; ^4^ Biochemistry of Plant‐Microbe Interactions, Institute of Biology Freie Universität Berlin Germany; ^5^ Institute for Macromolecular Chemistry University of Freiburg Germany

**Keywords:** EIN2, ethylene signaling, importin alpha superfamily, NLS‐motif, nucleocytoplasmic transport

## Abstract

Plant hormones are small molecules that modulate a plethora of growth and developmental pathways. Among these molecules, ethylene is known to modulate several important agronomical traits, including fruit ripening and senescence. However, the mechanisms, pathways, and processes of ethylene signaling from the receptors at the endoplasmic reticulum (ER) membrane to the transcriptional regulators in the nucleus remain to be elucidated. Here, we demonstrate that the importin alpha superfamily of nuclear transport receptors plays a pivotal role by transporting ethylene key regulator ETHYLENE INSENSITIVE 2 (EIN2) from the ER into the nucleus. Our findings show that *importin α* (*impα*) single‐ and triple‐mutant seedlings of *Arabidopsis thaliana* retain a normal ethylene response, as evidenced by the typical triple‐response phenotype observed in the presence of ethylene. *In vitro* and *in planta* interaction studies demonstrate that EIN2 is recognized as cargo by all nine IMPα isoforms, though with distinct affinities. Specifically, the binding studies reveal that IMPα1/2/3/4/7 are the most relevant isoforms for the nucleocytoplasmic transport of EIN2. Based on computational interaction predictions, we have identified potential binding modes and offer novel mechanistic insights into the interaction between the nuclear localization signal (NLS) motif of EIN2 and the IMPα superfamily. Our results provide novel insights into the mechanism by which the ethylene signal is transmitted from the ER membrane to the nucleus. The data pave the way for a more comprehensive understanding of the ethylene signaling pathway and the central role of EIN2.

## Graphical abstract

The plant hormone ethylene regulates plant growth, ripening, senescence, and stress responses. The hormonal signal transmission, from receptors at the ER membrane to the transcriptional regulators in the nucleus, is still not completely understood. We show that the IMPα superfamily is crucial for the transport of EIN2, a critical molecule in the ethylene pathway, from the ER to the nucleus, with all nine IMPα isoforms recognizing EIN2's C‐END as cargo.

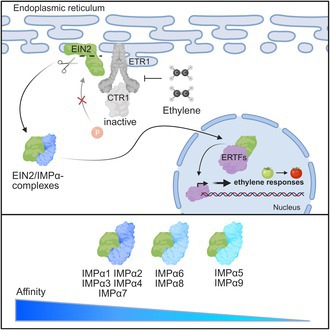

**Abbreviations**
ARM: armadillo repeat domain
At: *Arabidopsis thaliana*
BLI: biolayer interferometry
CER: cerulean
CTR1: CONSTITUTIVE TRIPLE RESPONSE1
EIN2 CEND: ethylene‐insensitive protein 2 C‐terminal cytosolic part
EIN2: ETHYLENE INSENSITIVE2
ER: endoplasmic reticulum
ETR: ethylene receptor
FLIM‐FRET: fluorescence lifetime microscopy and Förster's resonance energy transfer
FP: fluorescence polarization
GAF: cGMP‐specific phosphodiesterase, adenylyl cyclases, and FhlA
IBB: importin β binding domain
IMPα: importin α
IMPβ: importin β
*K*_d_
: dissociation constant
NLS: nuclear localization signal
NPC: nuclear pore complex
RAN: RAS‐RELATED NUCLEAR PROTEIN
YFP: YELLOW FLUORESCENCE PROTEIN

## Introduction

**Fig. 1:**
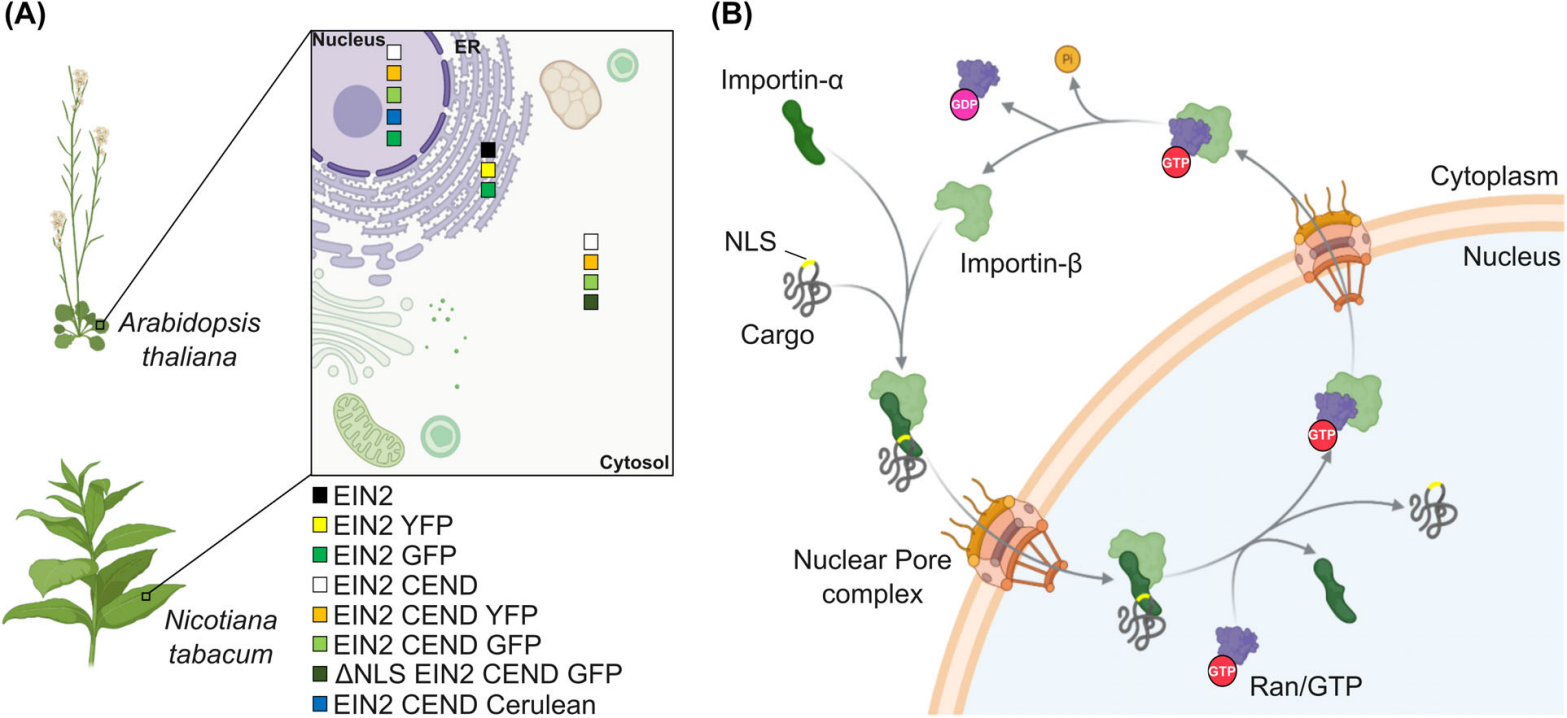
Mapping EIN2 and EIN2CEND localization in plant cells and the role of IMPα/β in nuclear import. (A) Localization of EIN2 (ETHYLENE INSENSITIVE 2) and EIN2 CEND (ETHYLENE INSENSITIVE 2 C‐terminal cytosolic part) based on reported *A. thaliana* and *N. tabacum in planta* experiments. (B) Simplified principle of the importin (IMP)α/β mediated nuclear import. During the IMPα/β mediated nuclear import, a cytoplasmic complex between IMPα, cargo nuclear localization signal (NLS), and IMPβ is formed. The IMPα/β‐cargo complex translocates into the nucleus through the nuclear core complex. In the nucleus, binding of Ras‐related nuclear protein/Guanosine‐triphosphate (RAN/GTP) to IMPβ triggers the dissociation of ternary import complexes and drives nuclear cargo release. IMPβ‐RAN/GTP complexes exit the nucleus. RAN/GTP hydrolysis and release of Ras‐related nuclear protein/Guanosine‐diphosphate (RAN/GDP) in the cytoplasm then free IMPβ for the next round of import. Both parts of the figure were created with BioRender.com.

The plant hormone ethylene plays a crucial role in plant growth and development, influencing processes such as fruit ripening, senescence, and adaptive responses of plants to a wide range of biotic and abiotic stresses [1, 2, 3, 4, 5]. Ethylene perception and responses are mediated by a family of integral membrane receptors (ETRs) localized at the endoplasmic reticulum (ER)–Golgi network [6, 7, 8]. The receptors, which in their functional state form homo‐ and heteromers at the membrane, act as negative regulators of the signaling pathway, following an inverse‐agonist model in which ethylene binding switches off downstream signal transmission [7, 9, 10, 11, 12, 13]. Receptor‐associated Raf‐like kinase CONSTITUTIVE TRIPLE RESPONSE1 (CTR1) and ER integral membrane protein ETHYLENE INSENSITIVE2 (EIN2) have been identified as pivotal downstream partners of the receptors and key elements in ethylene signal transmission [14, 15, 16]. Both interact with the ethylene receptors at the ER membrane [17, 18, 19], but for long it was unclear how the ethylene signal is transmitted from the ER to the nucleus. Bioinformatic sequence analysis revealed a putative nuclear localization signal (NLS) in the C‐terminus of EIN2 (residues 1261–1268), supporting the idea that the C‐terminal cytosolic part (amino acids 462–1294) is cleaved off the N‐terminal transmembrane part in response to the ethylene signal for translocation into the nucleus [20]. Under certain circumstances, the C‐terminus of EIN2 is localized in cytoplasmic P‐bodies [21, 22]. Multiple experimental studies provided mechanistic insights into the processes bridging ethylene transmission from the receptors located in the ER membrane to the nucleus [23, 24]. Additionally, binding studies revealed that the EIN2 NLS‐motif serves not only in nuclear localization but also provides a critical contact site for the GAF (cGMP‐specific phosphodiesterase, adenylyl cyclases, and FhlA) domain of the ETR receptors [25, 26]. EIN2 lacking the NLS motif shows strongly reduced affinity for the receptors. Synthetic peptides derived from the EIN2 NLS‐motif [25] also impede the interaction of EIN2 and the receptors. Notably, the corresponding peptides markedly impair ethylene responses in plants, such as fruit ripening or senescence [27, 28, 29, 30]. Further genetic and molecular studies on the ethylene signaling network revealed that the Raf‐like kinase CTR1 directly interacts with and phosphorylates EIN2 at multiple sites in the absence of the plant hormone [31, 32, 33, 34]. Conversely, the plant hormone inactivates CTR1, preventing CTR1‐dependent phosphorylation of EIN2 [33]. In turn, ER‐membrane localized EIN2 is proteolytically processed, and the C‐terminal end of EIN2, containing the NLS‐motif [20, 31], is translocated to the nucleus [31, 32, 33]. Deletion of the NLS retained EIN2 CEND in the cytoplasm, suggesting that the NLS sequence is responsible for CEND's nuclear localization [32]. In the nucleus, EIN2 CEND activates the transcription factor EIN3 [22, 32] and its paralogs by a mechanism that has yet to be fully elucidated, resulting in the transcription of ethylene response genes [35, 36]. The pathways and transport mechanisms of EIN2 CEND to and into the nucleus are not resolved yet. In addition to the previously reported localization of EIN2 in the ER membrane and EIN2 CEND in the nucleus [20, 31, 32, 33], several studies in *Arabidopsis thaliana* and *Nicotiana tabacum* have described alternative subcellular localization for both [17, 31, 32, 37, 38]. For instance, EIN2 has been observed in the nucleus [32], while EIN2‐CEND has been detected in the cytoplasm [31, 32, 37, 38] (Fig. 1A).

Macromolecules with a molecular weight larger than 40 kDa (e.g., EIN2 CEND (91.81 kDa)) require an active transport process for translocation across nuclear pore complexes (NPC) that span the double membrane of the nuclear envelope [39, 40, 41, 42]. Nuclear import receptors recognize their molecular cargos in the cytoplasm by distinct NLS sequence motifs, carry them across the NPC, and release them in the nucleus [43, 44]. Based on their recognition sequence, NLS motifs are classified as either monopartite (a single cluster of basic amino acids) or bipartite (two clusters of basic amino acids separated by a 10–12 amino acid linker) [45]. Sequence‐based prediction of NLSs in the cytosolic part CEND of the ethylene key regulator EIN2 reveals that the CEND contains mono‐ and bipartite NLSs [20], suggesting an interaction with the IMPα superfamily. In the classical nuclear import pathway, which is conserved from yeast to humans [46], a cytoplasmic complex is formed. Thereby, NLS motifs in the cargo proteins are recognized by the nuclear transport receptor importin α (IMPα) which, upon NLS binding, functions as an adaptor and bridges the interaction to the actual nuclear import carrier importin β (IMPβ), which mediates the passage of the ternary complex through the NPC [47, 48, 49] (Fig. 1B). Active transport of the IMPα‐IMPβ cargo complex across the NPC is mediated by the direct interaction of IMPβ and nucleoporin proteins in the NPC [50, 51]. Therefore, a crucial step in nuclear translocation is the specific recognition of the cargo protein by the IMPα receptor, as this complex formation ultimately determines which cargo is transported to the nucleus. RAS‐RELATED NUCLEAR PROTEIN (RAN), a small GTPase that exists in a GTP‐bound nuclear and GDP‐bound cytoplasmic state [52, 53, 54], controls the cargo binding to and release from the IMPα/β heterodimer. Binding of RAN·GTP to IMPβ in the nucleoplasm triggers the dissociation of ternary import complexes and drives nuclear cargo release [55, 56]. However, members of the IMPβ protein family can function independently of IMPα adapters to mediate nuclear cargo import [57, 58, 59]. Lu *et al*. [60] demonstrated that IMPβ can mediate nuclear traffic of EIN2 CEND in *Arabidopsis thaliana*.

Structural studies have shown that IMPα proteins of different origin show conserved architectures consisting of two functionally and structurally distinct domains [61, 62]. The C‐terminal armadillo repeat domain (ARM) provides distinct binding sites for cargo NLS motifs. Repeats 2–4 shape the major site, whereas repeats 6–8 form the minor NLS binding site [63, 64, 65, 66]. The major site is regarded as the high‐affinity binding site for canonical monopartite NLSs, whereas bipartite NLSs bind to both the minor and the major site [67]. The flexible auto‐inhibitory N‐terminal IMPβ binding domain (IBB) of IMPα contains a sequence related to bipartite NLSs, which competes with NLS cargo for binding to the ARM repeats and facilitates interaction with IMPα upon cargo binding as well as release of the cargo in the nucleoplasm [68, 69]. The *A*. *thaliana* genome encodes eight IMPα isoforms (IMPα1–IMPα7 and IMPα9) that contain both IBB domains and ARM repeats, as well as a single isoform (IMPα8) that contains only ARM repeats [70, 71].

Here, we demonstrate in cell biological, biophysical, and *in planta* studies that EIN2‐NLS peptides and EIN2 CEND interact with the IMPα superfamily. Our results provide novel mechanistic insights into how EIN2 CEND translocates from the ER membrane to the nucleus, shedding light on processes by which EIN2 integrates and bridges signaling from the ER membrane into the nucleus to trigger stress and developmental responses upon perception of the plant hormone ethylene.

## Results

### Involvement of individual members of the IMPα superfamily in plant ethylene response

**Fig. 2:**
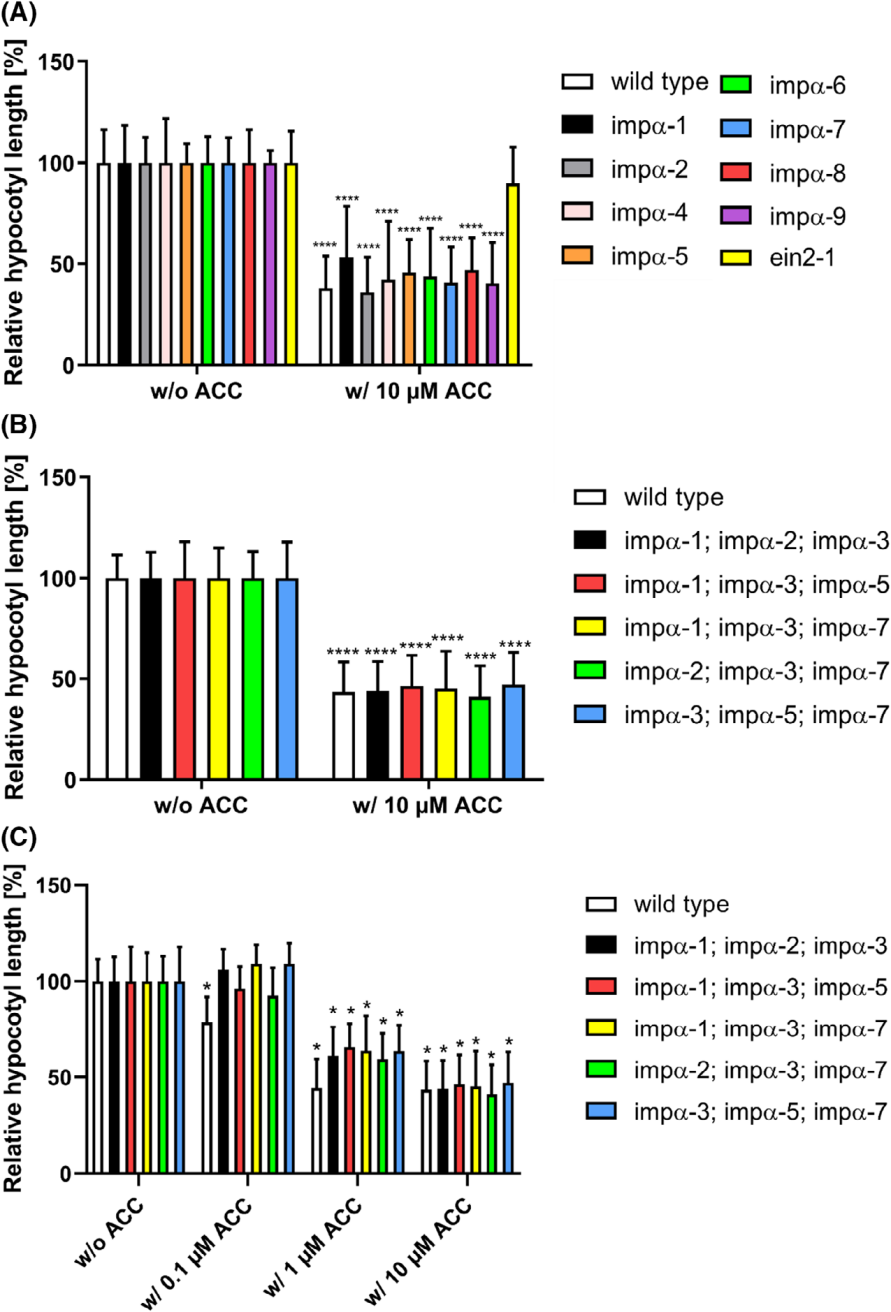
Triple response experiments with single and triple *impα* loss‐of‐function mutants. (A) Triple response experiments with single loss‐of‐function *impα* mutants and (B and C) with triple loss‐of‐function *impα* mutants. The wild‐type Col‐0 served as a positive control, while the mutant *ein2‐1* functioned as the negative control for the triple response. The *impα3* single mutant did not germinate. The hypocotyl length of each plant line in the absence of the ethylene precursor 1‐aminocyclopropane‐1‐carboxylic acid (ACC) (w/o ACC) was set to 100%, and the hypocotyl length of each line in the presence (w/) of ACC is displayed in percent relative to the respective untreated control. Bars represent the mean ± SD of pooled data from at least 10 seedlings per condition for the single *impα* mutants and at least 20 seedlings per condition for the triple *impα* mutants. For statistical analysis a two‐way ANOVA and Dunnett's multiple comparisons test was used. *P‐value < 0.05; ****P‐value < 0.0001 (w/o ACC vs. respective ACC concentration). In C), one asterisk equals four asterisks for a better overview.

In the presence of ethylene, dark‐grown seedlings display the characteristic phenotypic responses known as triple response [9, 10, 15, 72, 73]. We hypothesized that nucleocytoplasmic transport and nuclear import of the EIN2 cytosolic part (CEND), which bridges ethylene signal transduction from the ER membrane to the nucleus, are mediated by the IMPα‐IMPβ transport machinery. This hypothesis is supported by the previously identified NLS motif in the EIN2 C terminus. To investigate whether any of the IMPα isoforms are implicated in ethylene signaling, *A. thaliana* Col‐0 (wild‐type; positive control), single *loss‐of‐function* mutants of the IMPα isoforms (*impα1‐impα9*) obtained from the Nottingham Arabidopsis Stock Centre (NASC), and ethylene‐insensitive mutant *ein2‐1* (negative control) were grown in the dark in the presence of the ethylene precursor 1‐aminocyclopropane‐1‐carboxylate (ACC) and subsequently analyzed for their triple response. Fig. 2A illustrates that all single *impα* mutants besides *impα‐3*, which was excluded from the study due to seed germination issues, showed the typical triple response, similar to the wild‐type Col‐0, with a 54–64% decrease in hypocotyl length for Col‐0 and the *impα* mutants. In contrast, the triple response was not observed in the *ein2‐1* loss‐of‐function control, which showed only a 10% decrease in hypocotyl length. The findings on the single *impα* mutants indicate that nucleocytoplasmic transport and nuclear import of EIN2 CEND cannot be attributed to a single IMPα isoform, possibly because of functional redundancy among the IMPα isoforms. This is in line with the high homology and large conservation among the IMPα isoforms, with 42% identity and 61% conservation in the amino acid sequence of canonical IMPα1‐8, suggesting that other family members compensate for the loss of one IMPα isoform. IMPα9 possesses here a special position because of a sequence identity <30%. A sequence alignment within the *Arabidopsis thaliana* IMPα family is shown in Fig. S1.

To gain further insights into the role of the IMPα superfamily for ethylene signaling, we studied multiple higher order *IMP*α loss‐of‐function mutants [74] (Fig. 2). The triple mutants *impα‐1;impα‐2;impα‐3*, *impα‐1;impα‐3;impα‐5*, *impα‐1;impα‐3;impα‐7*, *impα‐2;impα‐3;impα‐7*, and *impα‐3;impα‐5;impα‐7* all showed the typical triple response phenotype when grown in the dark and exposed to the ethylene precursor ACC (Fig. 2B). The decrease in hypocotyl length of the triple mutants ranged from 53% to 59%, which is comparable to the decrease observed for the single *impα* mutants (Fig. 2A). These results from the analysis of multiple loss‐of‐function mutants support the hypothesis that the IMPα isoforms function redundantly in the nuclear import of EIN2. To check for potential differences in ethylene sensitivity, we supplemented the experiments from Fig. 2B with 0.1 and 1 μm ACC (Fig. 2C). The wild‐type Col‐0 exhibited a relative reduction in hypocotyl length of 21% even at 0.1 μm ACC, whereas all triple mutants remained unaffected, showing a maximum reduction of 7.5%. At 1 μm ACC, the decrease in wild‐type seedlings (55.5%) was comparable to that observed at 10 μm ACC (56.5%). In contrast, the hypocotyl length reduction in the triple mutants ranged from 34% to 41% at 1.0 μm and from 53% to 59% at 10 μm ACC as previously noted. These findings indicate that the mutation of three IMPα isoforms leads to diminished sensitivity in ethylene perception, supporting the pivotal role of the IMPα superfamily in ethylene signaling. However, no differences in ethylene sensing were observed among the various triple mutants, further reinforcing the hypothesis of IMPα redundancy in EIN2 transport. The deletion of an excessive number of IMPα isoforms is likely to have detrimental effects on regular plant growth and development, which has already been observed in the triple mutants (*impα‐1;impα‐2;impα‐3*) [74]. Due to the pivotal role of the IMPα‐IMPβ machinery in the nuclear transport of a large number of cargo proteins, we adopted an alternative strategy to investigate the role of IMPα proteins in plant ethylene signal transduction by testing their physical interaction with the ethylene key regulator EIN2.

### EIN2 interacts with and binds to all members of the IMPα superfamily

**Fig. 3:**
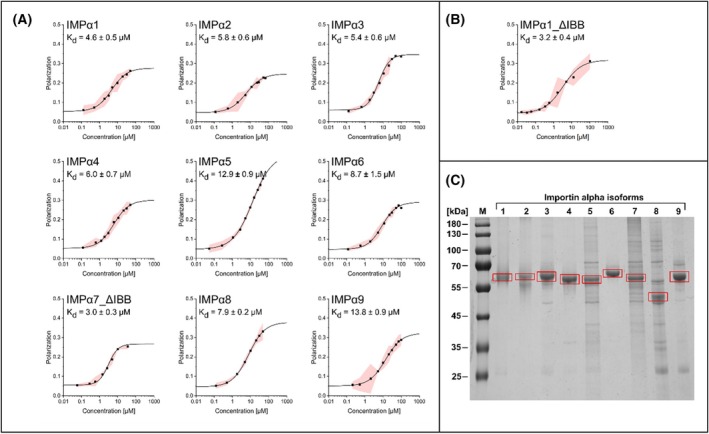
Fluorescence polarization (FP) measurements of IMPα isoforms 1–9 with the TAMRA‐NOP‐1 peptide as a proxy for EIN2 CEND. For importin (IMP)α7 a variant lacking the N‐terminal importin β binding (IBB) domain was employed as expression and purification of full‐length IMPα7 was not successful. The polarization is plotted against the protein concentration in μm. Data points represent mean values with 95% confidence intervals based on three independent measurements. Dissociation constants (*K*_d_) were determined by using the Logistic Fit function in Origin. (A) *K*_d_ value determination for IMPα1‐9 and IMPα7_ΔIBB. (B) *K*_d_ value determination for IMPα1_ΔIBB. Data points represent mean values with 95% confidence intervals based on three independent experiments (*n* = 3). The SDS‐PAGE for all purified isoforms is depicted in (C). The red boxes indicate the purified IMPα isoforms.

To clarify whether EIN2 is a potential cargo protein of the IMPα superfamily, all nine IMPα isoforms of *A. thaliana* were expressed recombinantly in *E. coli* and purified to homogeneity from the bacterial host (Fig. 3C). Interaction of purified IMPα proteins was probed by fluorescence polarization (FP) measurements with a set of EIN2‐NLS peptides and shortened EIN2 CEND (Fig. 3A,B, Table S1). FP takes advantage of the fact that a fluorescent molecule, when excited by polarized light, emits light with a polarization level inversely related to its molecular rotation rate. This feature makes FP a valuable tool for studying the binding of small labeled ligands or peptides to larger proteins and serves as the basis for both direct and competitive binding assays [75].

In these studies, we initially examined the interaction between TAMRA‐NOP‐1, a peptide that corresponds to the NLS motif of EIN2 and is N‐terminally labeled with the fluorescent dye 5‐TAMRA (5‐carboxytetramethylrhodamine), and the nine *Arabidopsis* IMPα proteins. The results of these binding studies revealed that binding occurred for all nine IMPα isoforms (Fig. 3A). While the majority of the IMPα proteins demonstrated comparable affinities for TAMRA‐NOP‐1, with dissociation constants (*K*_d_s) in the low micromolar range (3–6 μm), isoforms IMPα5 and IMPα9 showed lower affinities, with *K*_d_ values of 12.9 ± 0.9 μm and 13.8 ± 0.9 μm, respectively. Binding affinities of IMPα6 and IMPα8 were only slightly diminished compared to the majority, with *K*_d_ values of 8.7 ± 1.5 μm and 7.9 ± 0.2 μm, respectively. In conclusion, these findings indicate that all nine members of the *A. thaliana* IMPα superfamily are capable of binding EIN2 *in vitro* as a cargo and support a physiological role of the IMPα‐EIN2 interaction. The IMPα binding affinities indicate that they are not equally crucial for the binding and transport of EIN2 to and into the nucleus or for cytoplasmic trapping of EIN2 CEND. Previous binding studies between several NLS peptides and *A. thaliana* IMPα3 lacking the auto‐inhibitory IBB domain [76] have observed affinities in the low micromolar range (0.7–5.4 μm). Based on our findings, the IMPα superfamily can be classified into the following three groups: IMPα1/2/3/4/(7), IMPα6/8, and IMPα5/9. Given the low dissociation constants of the IMPα‐EIN2‐NLS complexes, isoforms IMPα1/2/3/4/(7) seem to be most relevant for EIN2 nucleocytoplasmic transport.

**Fig. 4:**
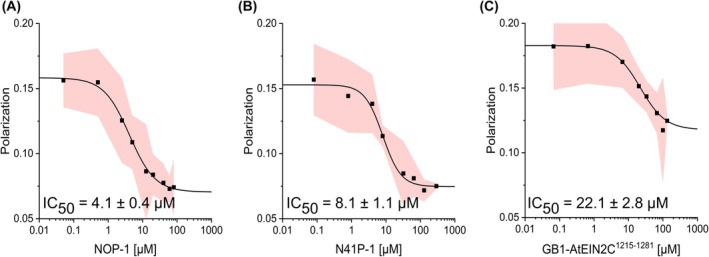
Competitive fluorescence polarization (FP) measurements with EIN2 peptides of different lengths against TAMRA‐NOP‐1 bound to IMPα4. The tested EIN2 peptides included (A) NOP1, (B) N41P‐1, and (C) GB1‐*At*EIN2C^1215–1281^. The concentration of IMPα4 (6 μm) was selected based on the previously determined *K*_d_ value of TAMRA‐NOP‐1 (TAMRA, Carboxytetramethylrhodamin). Polarization was plotted against the competitor concentration in μm. Data points represent mean values with 95% confidence intervals based on three independent experiments (*n* = 3). IC_50_‐values were determined using the Logistic Fit function in Origin.

To substantiate the hypothesis that EIN2 CEND serves as a cargo recognized by the IMPα superfamily and to confirm the NOP‐1 binding specificity, competitive FP measurements were performed. In these studies, TAMRA‐NOP‐1 bound to IMPα4 was selected as a representative interaction of the IMPα superfamily. TAMRA‐NOP‐1 was subsequently displaced by the addition of selected EIN2 CEND‐NLS constructs of different lengths. Two of the constructs (NOP‐1, EIN2 residues: 1262–1269; N41P‐1, EIN2 residues: 1229–1269) were produced synthetically, while CEND construct GB1‐*At*EIN2C^1215–1281^ containing a GB1‐tag to enhance solubility was produced recombinantly in *E. coli*. The binding curves shown in Fig. 4 clearly demonstrate that all three EIN2 CEND‐NLS constructs were able to displace previously bound TAMRA‐NOP‐1, as indicated by a decrease in fluorescence polarization. Notably, the affinity of the different CEND peptides for IMPα4 decreased with increasing amino acid length. NOP‐1 displayed the highest affinity of all EIN2‐NLS probes, with a *K*_d_ of 4.1 ± 0.4 μm (Fig. 4A), which closely aligns with the observed affinity of IMPα4 for TAMRA‐NOP‐1 (~6 μm). With the extended EIN2‐NLS probe N41P‐1 as competitor, a *K*_d_ of 8.1 ± 1.1 μm was observed (Fig. 4B), while the most extended CEND probe GB1_*At*EIN2C^1215–1281^ showed binding with a *K*_d_ of 22.1 ± 2.8 μm (Fig. 4C). The displacement of prebound TAMRA‐NOP‐1 by all EIN2‐NLS CEND probes unequivocally confirms the specific binding of the EIN2‐NLS core motif (NOP‐1) and longer EIN2 CEND constructs. These results provide further support for the hypothesis that EIN2 CEND serves as a cargo protein of the IMPα superfamily to be transported from the ER to the nucleus via the IMPα‐IMPβ machinery.

Next, we used biolayer interferometry (BLI) with an Octet K2 instrument (Fortè Bio, Dallas, USA) to substantiate and further delineate the binding interactions between the IMPα superfamily and the EIN2‐NLS motif. BLI measures slight changes in reflected light at a sensor surface upon the formation of molecular complexes. Specifically, it monitors interference patterns of white light, which are reflected from two different surfaces: an internal reference layer and a layer of biomolecules immobilized on a biosensor tip [77].

**Fig. 5:**
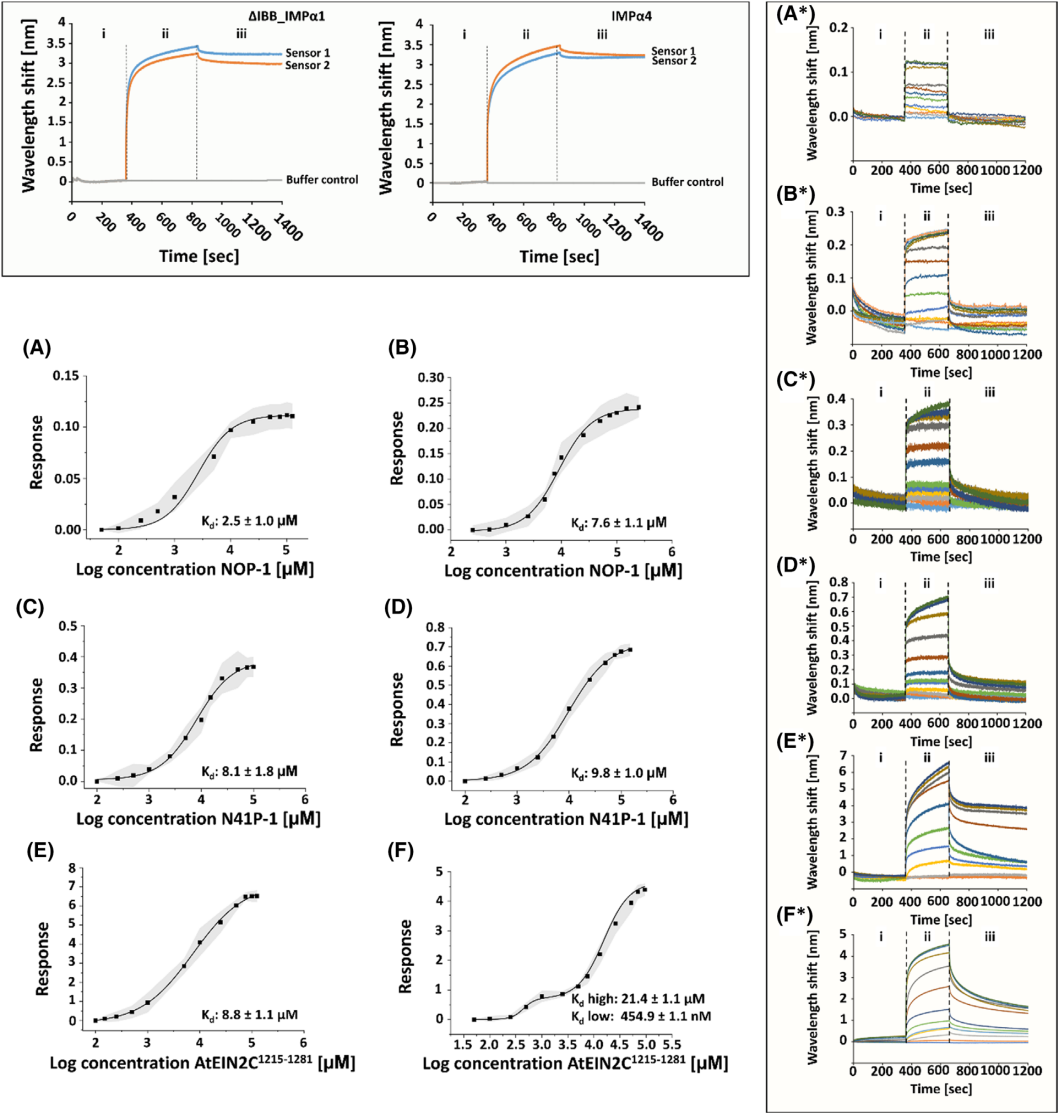
Binding studies and dissociation constant (*K*_d_) determination of different ligands to ΔIBB_IMPα1 and IMPα4 using BLI sensorgrams. The box on the top of the figure shows real‐time sensorgram traces of the Biolayer Interferometry (BLI) protocol that uses streptavidin‐coated biosensors. (i) baseline, (ii) loading with biotinylated ΔIBB_IMPα1 and IMPα4 (IBB, importin β binding domain; IMP, importin), (iii) running buffer baseline step after loading. A*–F* show real‐time sensorgram traces of the BLI experiments that use streptavidin‐coated biosensors, which were loaded with biotinylated ΔIBB_IMPα1 and IMPα4. Raw traces are shown for ΔIBB_IMPα1‐NOP‐1 (A*), ‐N41P‐1 (C*), ‐*At*EIN2C1215–1281 (E*), for IMPα4‐NOP‐1 (B*), ‐N41P‐1 (D*), and ‐*At*EIN2C1215–1281 (F*). A BLI protocol was used as follows: (i) running buffer baseline step, (ii) association, and (iii) dissociation. (Related to Table 1). Based on the BLI real‐time sensorgram traces, the dissociation constant (*K*_d_) of different ligands to ΔIBB_IMPα1 and IMPα4 was determined. The data are shown as dose response curves. The *K*_d_ values are implemented in the corresponding graphs. *K*_d_ values were determined for ΔIBB_IMPα1‐NOP‐1 (A), ‐N41P‐1 (C), ‐*At*EIN2C^1215–1281^ (E), and for IMPα4‐NOP‐1 (B), ‐N41P‐1 (D), and ‐*At*EIN2C^1215–1281^ (F). Data points represent mean values with 95% confidence intervals based on three independent BLI experiments (*n* = 3). (Related to Table 1).

In these binding assays, IMPα1 lacking the IBB domain (ΔIBB_IMPα1) and IMPα4 were selected as representatives of the IMPα superfamily. Prior to their interaction studies with different EIN2 CEND‐NLS probes, both importins were biotinylated with the Biotin‐PEG4‐NHS ester Protein Labeling Kit from Jena Bioscience (Jena, Germany) and immobilized onto streptavidin‐coated biosensor tips (Fig. 5).

Following their immobilization, the loaded biosensor tips were dipped into wells containing NOP‐1, N41P‐1, and GB1‐*At*EIN2C^1215–1281^, respectively, which served as EIN2 CEND‐NLS probes of varying lengths and complexities. The resulting association and dissociation curves for serial dilutions of NOP‐1, N41P‐1, and GB1‐*At*EIN2C^1215–1281^ with ΔIBB_IMPα1 and IMPα4 are presented in Fig. 5. The analysis of the binding kinetic data and the processing of BLI sensorgrams by the implemented Octet® Analysis Studio Software revealed dissociation constants (*K*_d_s) in the low μm range (2.5–21.4 μm) for NOP‐1, N41P‐1, and GB1‐*At*EIN2C^1215–1281^ (Table 1; Fig. 5A–E), with the basic EIN2‐NLS probe (NOP‐1) showing the highest affinity (ΔIBB_IMPα1: 2.5 ± 1.0 μm and IMPα4: 7.6 ± 1.1 μm as shown in Fig. 5A,B). These findings agree well with the previously determined binding constants measured by FP.

**Table 1:** Binding parameters for the interaction of IMPα representatives ΔIBB_IMPα1 and IMPα4 with EIN2‐NLS CEND probes NOP‐1, N41P‐1, and GB1‐*At*EIN2C^1215–1281^.

Ligand	ΔIBB_IMPα1	
	*K*_d_ + SD	Hill coefficient
GB1‐*At*EIN2C^1215–1281^	8.8 ± 1.1 μm	0.94 ± 0.06
N41P‐1	8.1 ± 1.8 μm	1.25 ± 0.11
NOP‐1	2.5 ± 1.0 μm	1.03 ± 0.09

Ligand	IMPα4	
	*K*_d_ + SD	Hill coefficient
GB1‐*At*EIN2C^1215–1281^	*K*_d_ high: 21.4 ± 1.1 μm *K*_d_ low: 454.9 ± 1.1 nm	0.15 ± 0.02
N41P‐1	9.8 ± 1.0 μm	1.09 ± 0.05
NOP‐1	7.6 ± 1.1 μm	1.15 ± 0.04

Curve fitting of the BLI sensorgrams was tested with different binding models. The best fit was obtained with a 1:2 heterogeneous ligand binding model (data not shown). This model assumes analyte binding at two independent ligand sites at the IMPα immobilized on the biosensor surface. Furthermore, each ligand site binds the analyte independently and with a different rate constant. Notably, the dose response curve for the GB1‐*At*EIN2C^1215–1281^ interaction with IMPα4 showed a clear biphasic sigmoidal curve (Fig. 5F). Based on this observation, a low and high *K*_d_ were determined, with the low *K*_d_ in the medium nm range and the high *K*_d_ in the higher μm range (Table 1). In this case, the second *K*_d_ in the higher μm range may be attributed to the binding of the GB1‐tag.

### Structural basis for the interaction of the IMPα superfamily with EIN2‐NLS motif

**Fig. 6:**
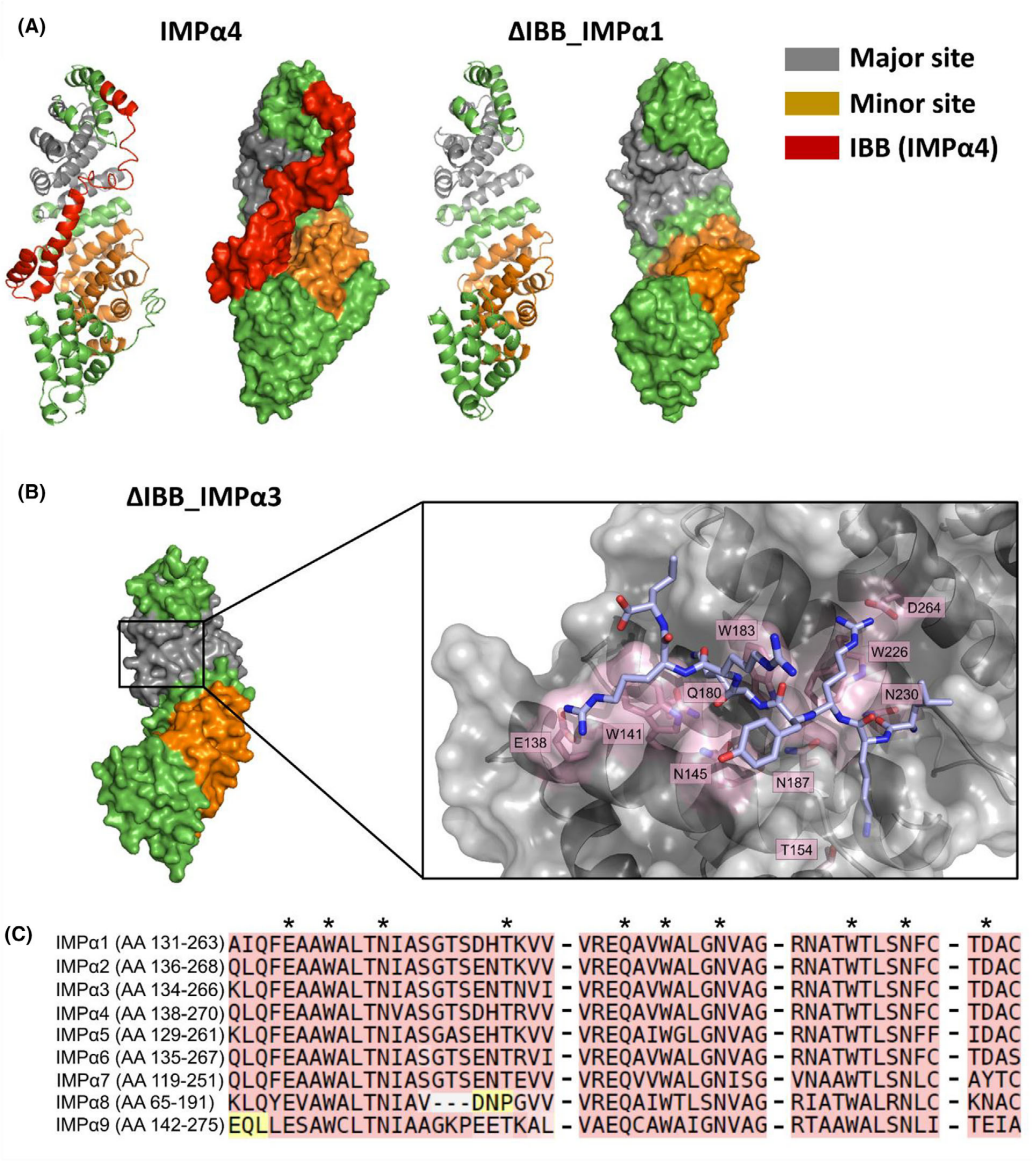
Structural modeling of IMPα isoforms and predicted NOP‐1 interaction with ΔIBB_IMPα3. (A) The structural models of ΔIBB_IMPα1 and IMPα4 (IBB, importin β binding domain; IMP, importin) were generated using the I‐tasser online server. Structures are depicted in both ribbon and surface views. The major nuclear localization sequence (NLS) binding site, the minor NLS binding site, and the IBB domain are highlighted in gray, orange, and red, respectively. (B) Predicted binding mode of NOP‐1 with IMPα3 lacking the IBB domain at the major NLS site. Binding position of the EIN2‐NLS probe NOP‐1 (light blue and in sticks) in the IMPα3 armadillo repeat (ARM) domains (gray; PDB code: 4TNM) as revealed by AlphaFold2 [78] modeling. Residues involved in the interaction are highlighted in pink and labeled with their one‐letter amino acid code and position. Oxygen and nitrogen atoms are marked in red and blue, respectively. (C) Alignment of amino acid sequences of the nine IMPα isoforms predicted to be involved in NOP‐1 binding, using T‐COFFEE [79]. Residues depicted in A are marked with asterisks. T‐COFFEE highlights residues with a good alignment in pink and residues with an averaged alignment in yellow.

Homology modeling of ΔIBB_IMPα1 and IMPα4 was performed to demonstrate the position of the IBB domain in the structure and its effect on the major and minor NLS binding site (Fig. 6A). To further localize EIN2‐NLS binding to the IMPαs, structural models of ligand–protein complexes of the IMPα3 core structure (PDB code: 4TNM) lacking the IBB domain with the NOP‐1 peptide were generated with AlphaFold2 [78] using *ColabFold* 1.5.5. The resulting models suggest that NOP‐1 is bound by the major NLS binding site of IMPα3, which is the preferred binding site for canonical monopartite NLS. Key interactions of IMPα3 with the EIN2‐NLS CEND probe were predicted at residues E138, W141, N145, T154, Q180, W183, N187, W226, N230, and D264 (Fig. 6B). To investigate whether any of these pivotal interactions could account for the differences in the EIN2‐NLS binding affinities observed in our *in vitro* binding studies among the various IMPα isoforms, a multiple sequence alignment using T‐COFFEE [79] with all nine *A. thaliana* IMPα isoforms was done.

As illustrated in Fig. 6C, the alignment reveals that IMPα1‐6 show no variation at these pivotal interactions, IMPα7 and IMPα9 display one alteration each (IMPα7: D264Y; IMPα9: D264E), while IMPα8 has two distinct changes (IMPα8: T154P and D264N). In conclusion, it is unlikely that these changes cause the observed differences in binding affinities. However, based on the assumption that the auto‐inhibitory N‐terminal IBB domain competes for NLS binding at the major rather than at the minor NLS binding site [59, 80, 81] and given the incomplete match of the NOP‐1 NLS‐sequence (LKRYKRRL) with any of the six NLS classes defined by Kosugi *et al*. [82], it is reasonable to assume that NOP‐1 may in fact bind at the minor site of IMPα proteins. Pang and Zhou demonstrated that residues G323, D325, W357, N361, E396, W399, and N403 are involved in the binding of non‐canonical monopartite NLS at the minor site of mouse IMPα lacking the IBB domain [83]. Based on the sequence alignment and comparison of the various IMPα isoforms of *A. thaliana* with respect to the aforementioned residues involved in the minor site‐specific binding, these isoforms can be classified into four distinct groups: IMPα1‐4 and IMPα6‐7 (no alternation with regard to the NLS‐minor binding site), IMPα5 (D319S), IMPα8 (D319H), and IMPα9 (G317V; D319P and W394Y) (Fig. S2). This classification aligns well with the results of our *in vitro* binding studies and the overall phylogenetic clustering of the nine isoforms [70, 84]. The only exception of this classification is IMPα6, which shows lower binding affinity than the other isoforms of the same category. The highest affinity was observed for IMPα1 (Fig. 3B) and IMPα7 lacking the IBB domain (Fig. 3A), with affinities of 3.2 ± 0.4 μm and 3.0 ± 0.3 μm, respectively. However, these affinities differ only slightly from those of full‐length IMPαs showing low micromolar affinities. Accordingly, the results from our binding and computational studies can be interpreted in two ways. First, NOP‐1 may bind preferentially at the minor binding site, regardless of whether the IBB domain is present or absent. Secondly, the small EIN2‐NLS probe NOP‐1 may bind preferentially at the major binding site, preventing auto‐inhibition of the IBB domain with respect to NOP‐1 binding. This hypothesis is further supported by the fact that reduced or absent auto‐inhibition was observed for IMPα3 and IMPα4 in binding and genetic studies, respectively [76, 85]. Further experiments are required to clearly identify the EIN2 CEND‐NLS binding site in the IMPα superfamily. This may entail co‐crystallization of IMPαs with NOP‐1 or chemical crosslinking of IMPαs with photoactivable NOP‐1.

### The IMPα superfamily interacts with EIN2 CEND *in planta* and mediates its nucleocytoplasmic transport

To further investigate the interaction between members of the IMPα superfamily and EIN2 *in planta*, we used *in vivo* fluorescence lifetime microscopy and Förster's resonance energy transfer (FLIM‐FRET). Accordingly, the C‐terminal part of EIN2 (EIN2C^479–1294^) [17] was fused to the fluorescent protein Cerulean (CER), serving as a donor, and the IMPα isoforms (IMPα1‐7) were fused to YELLOW FLUORESCENCE PROTEIN (YFP), serving as acceptors. Transcriptome analysis on different *A. thaliana* tissues and aging plants demonstrated that IMPα8‐9 are expressed in low amounts compared with IMPα1‐7, especially during senescence of *A. thaliana* [86, 87, 88].

**Fig. 7:**
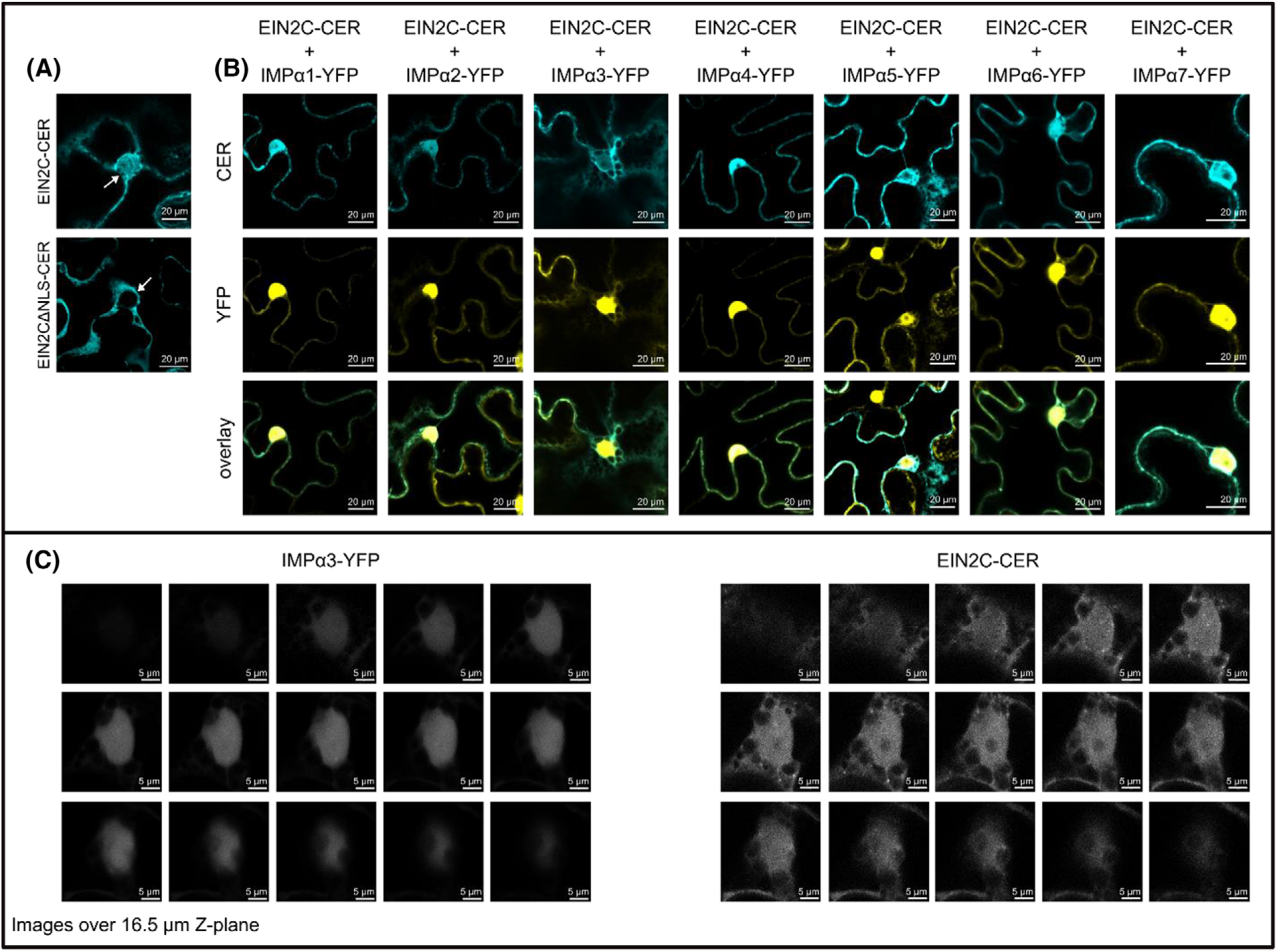
Confocal fluorescence microscopy of the C‐terminal end of the ethylene key regulator EIN2 and *Arabidopsis thaliana* IMPα superfamily members IMPα1‐7 in transiently transformed *N. benthamiana* epidermal leaf cells. (A) Confocal images of EIN2C^479–1294^ (Ethylene‐insensitive protein 2 C‐terminal cytosolic part^479^‐^1294^) and EIN2^479‐1294^ΔNLS (Ethylene‐insensitive protein 2^479^–^1294^Δnuclear localization signal) fused to Cerulean (Cer). White arrows indicate the nucleus. All scale bars equal 20 μm. (B) Confocal images of co‐expressed EIN2C^479‐1294^‐CER and *At*IMPα1‐7 (*Arabidopsis thaliana* importinα1‐7) fused to yellow fluorescence protein (YFP). Images show the emission channel for Cerulean (upper panel), YFP (middle panel), and an overlay of both fluorescence signals (lower panel). All scale bars equal 20 μm. (C) Representative images over 16.5 μm Z‐axis with IMPα3‐YFP and EIN2C‐CER. All scale bars equal 5 μm.

Consequently, IMPα8 and 9 were excluded from these experiments. The other constructs were transiently expressed in *N. benthamiana* epidermal leaf cells. EIN2C^479–1294^ localized in the nucleus, ER, and cytoplasm as previously shown by Bisson and Groth [25], whereas the EIN2C^479–1294^ΔNLS variant was only visible in the cytoplasm and ER and absent from the nucleus (Fig. 7). Co‐expression of EIN2C^479–1294^ and the IMPa isoforms revealed co‐localization of both proteins in the cytoplasm and the nucleus (Fig. 7A,B), which was further confirmed by imaging over 16.5 μm in the Z‐plane with IMPα3‐YFP as a representative (Fig. 7C). Nucleoli are clearly visible and do not display signal from EIN2 CEND‐CER. The EIN2 CEND‐CER signal is evenly distributed around the nucleolus, overlapping with the signal from IMPα3‐YFP, suggesting the signal is coming from the nucleus and not from the ER that is attached to the nucleus.

**Fig. 8:**
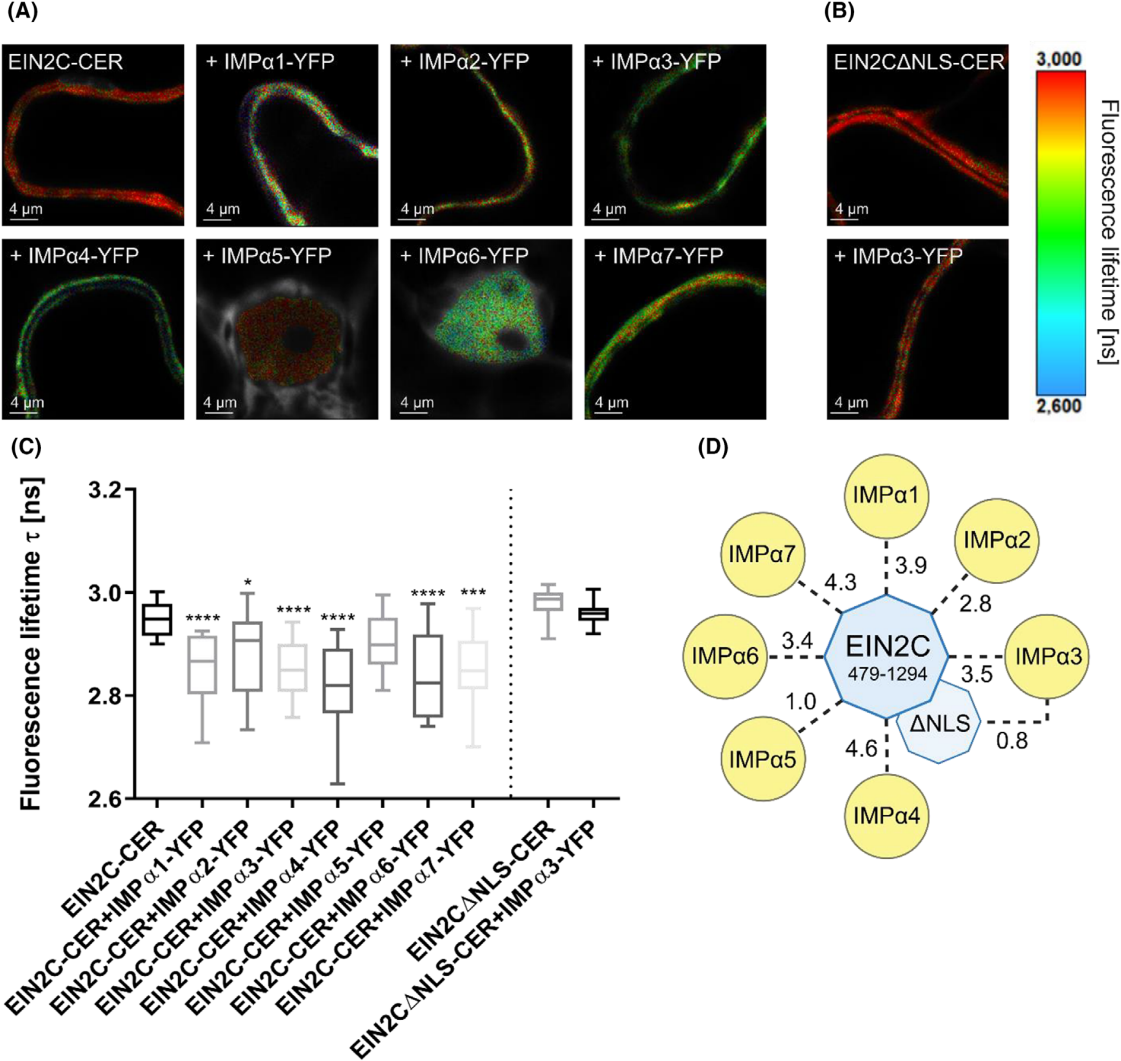
*In planta* FLIM‐FRET experiments using EIN2C^479–1294^ fused to Cerulean and seven IMPα isoforms fused to YFP. The *in planta* experiments were carried out in *N. benthamiana* epidermal leaf cells. EIN2C^479–1294^ fused to Cerulean (CER) was used as donor and the importin (IMP) α isoforms fused to yellow fluorescent protein (YFP) were used as acceptor. (A) Confocal images showing average fluorescence lifetime of the donor in the absence and presence of the different acceptor proteins IMPα1‐IMPα7‐YFP either in the cytoplasm or the nucleus. (B) Average fluorescence lifetime in the cytoplasm of EIN2C^479–1294^ΔNLS (Ethylene‐insensitive protein 2479–1294Δnuclear localization signal) in the absence and presence of the acceptor protein IMPα3‐YFP served as negative control. Scale bars indicate 4 μm. (C) Boxplots show the median (line), 25‐75th percentile (box) and 10‐90th percentile (whiskers) of pooled data from at least 18–20 measurements for the indicated protein (co)‐expressions. Values outside the 10‐90th percentile are not shown. Statistical analysis: Kruskal‐Wallis and Dunn's multiple comparisons test. *P‐value <0.05; ***P‐value <0.001; ****P‐value <0.0001 (donor only vs. donor + acceptor). The statistical analysis of the EIN2C^479–1294^ΔNLS‐CER variant with IMPα3‐YFP was performed by using a t‐test. (D) Graphical visualization of mean förster resonance energy transfer (FRET) efficiencies calculated for each donor‐acceptor pair which are indicated by dashed lines. Numbers next to the dashed lines reflect FRET efficiencies. The EIN2C^479–1294^ΔNLS‐CER fusion protein was only measured with IMPα3‐YFP and served as a negative control. Created in BioRender (2024) https://BioRender.com/l78k942.

While EIN2C^479–1294^ was predominantly localized in the cytoplasm, the IMPα isoforms were mainly found in the nucleus. The presence of IMPα1‐4‐YFP, IMPα6‐YFP, and IMPα7‐YFP resulted in a significant reduction in the average fluorescence lifetime of the EIN2C^479‐1294^‐CER donor, indicating interaction between EIN2 and the respective IMPα protein. When IMPα5‐YFP served as the acceptor, the average donor lifetime was only slightly, but not significantly reduced (Fig. 8A–C).

FRET efficiencies ranged between 2.8–4.6%. Samples containing IMPα4 demonstrated the highest FRET efficiency (4.6%), while samples with IMPα5 exhibited a FRET efficiency of only 1% (Fig. 8D). Given that nuclear import of the EIN2C^479–1294^ΔNLS variant is abolished [25], we then tested whether this is due to an impaired interaction with IMPα proteins. Co‐expression of the IMPα3‐YFP acceptor did not result in a reduction of the fluorescence lifetime of EIN2^479–1294^ΔNLS‐CER, indicating that no interaction occurred and suggesting that the EIN2^479–1294^ΔNLS variant is not imported into the nucleus (Fig. 8). These findings strongly support a physiological role of the IMPα superfamily in nucleocytoplasmic transport of EIN2, which is essential for mediating ethylene responses. Additionally, there is a notable redundancy in terms of IMPα isoforms that interact with EIN2, possibly to guarantee efficient nuclear import of this key regulator of ethylene‐mediated plant responses.

## Discussion

The mechanisms, pathways, and processes by which EIN2, the central switch of the ethylene response pathway, integrates and bridges signaling of the plant hormone from the ER membrane to the nucleus have been studied in‐depth and led to important insights in plant hormone signal transduction. While the generally accepted EIN2 ‘cleave and shuttle’ model plausibly explains how the ethylene signal is transmitted from the ER to the nucleus, the specific elements and structures of the nuclear import pathway of the processed EIN2 C‐terminus were entirely unknown. Based on the canonical NLS‐sequence found in the EIN2 C‐terminus and the impact of this sequence on nuclear import of CEND [31, 32], our study hypothesized that transport of the released EIN2 CEND from the cytoplasm into the nucleus is mediated by the IMPα‐IMPβ transport machinery. A study from Lu *et al*. [60] elucidated that the nuclear trafficking of *A. thaliana* EIN2 CEND can be induced by importin β1 (IMPβ1), either by ethylene treatment or by green peach aphid infestation. During the NLS‐dependent protein trafficking from the cytoplasm into the nucleus, mediated by the IMPα‐IMPβ transport machinery, the NLS on the cargo proteins is recognized by the IMPα subunit, which in turn is recognized by the IMPβ subunit [89]. An AlphaFold model of the *A. thaliana* IMPα1‐IMPβ1‐NOP‐1 demonstrates that the NLS sequence interacts with IMPα1 and that IMPβ1 may interact with regions of bigger cargo proteins like EIN2 CEND (Fig. S3). The identification of the binding region between IMPβ1 and EIN2 CEND is so far unknown and needs further investigations.

To ascertain whether the cargo recognition adapter module of this machinery (IMPα) is implicated in the recognition and binding of EIN2 CEND, we initially studied the well‐documented triple response, a set of morphological changes to dark‐grown seedlings in response to ethylene, in a range of single and triple *imp*α loss‐of‐function *Arabidopsis* mutants. Our results show that all studied *impα* single and triple mutants show the characteristic triple response phenotype when grown in the presence of the ethylene precursor ACC. This excludes the possibility that a single or a combination of three of the nine IMPα isoforms in *Arabidopsis* is specifically or exclusively responsible for EIN2 CEND nucleocytoplasmic transport, indicating a high level of functional redundancy of the IMPα isoforms for EIN2 CEND recognition and binding. Given the crucial role of the IMPα superfamily in the recognition and nucleocytoplasmic transport of a vast array of cargo proteins, as evidenced by the dwarf growth type of the *impα1*;*impα2*;*impα3* triple mutant [74], we reasoned that further deletion of additional members of the IMPα superfamily would result in severe developmental defects or plant lethality, thereby precluding triple response analysis as a means to study the impact of the IMPα superfamily on EIN2 CEND recognition and nucleocytoplasmic transport. Additionally, loss‐of‐function mutants of IMPβ1 and other IMPβ family proteins (e.g., HASTY and PAUSED) have shown strong effects on ethylene‐enhanced expression of defense mechanisms, plant growth, and development [60, 90, 91, 92]. This suggests that the IMPβ proteins may be involved in the nucleocytoplasmic transport of several pathways.

The outcome of our phenotypic studies led us to adopt a different approach to address the potential interactions of EIN2 CEND with members of the IMPα superfamily. We used purified recombinant IMPα1‐9 proteins with defined EIN2‐NLS probes for *in vitro* binding studies. The results of these studies demonstrated that EIN2‐NLS is recognized by most members of the IMPα superfamily at low micromolar affinity. Prior studies on purified IMPα from yeast, mammals, and plants [65, 93, 94, 95, 96] have demonstrated that functional canonical NLS have binding affinities up to 1 μm [97]. In contrast, binding studies between IMPα3 from *A. thaliana* lacking the IBB domain and NLS peptides [76] showed affinities in the low micromolar range, which is comparable to the affinities observed in our binding studies of the various *Arabidopsis* IMPα isoforms. The discrepancy to the previously reported sub‐micromolar affinities is likely due to the different techniques used to study biomolecular interactions. Affinities in the nanomolar range were determined by surface‐bound assays, including plate binding assays [65, 94, 96] and surface plasmon resonance (SPR) [95]. Conversely, affinities in the low micromolar range were obtained by isothermal calorimetry (ITC) [76] or, as in our case, by fluorescence polarization (FP) measurements. The FP‐based micromolar affinities for EIN2‐NLS are further corroborated by BLI measurements on immobilized IMPα isoforms with EIN2 CEND‐NLS probes of varying length and complexity, which revealed a decrease in affinity upon increasing sequence length of the NLS probe (*K*_d_ NOP‐1 < N41P‐1 < GB1_*At*EIN2^1215–1281^). The BLI measurements on the N‐terminal truncated IMPα1 further confirm the previously known competitive effect of the N‐terminal IBB domain for NLS binding. For EIN2‐NLS, this effect becomes more pronounced with increasing length, reduced flexibility of the NLS probe, and is most evident for GB1‐*At*EIN2C^1215–1281^. The second, high‐affinity binding observed in the sub‐micromolar range for the EIN2 CEND GB1‐fusion may be attributed to immobilization effects of the importin, as no such affinity was observed in the FP measurements with the various IMPαs in solution.

In order to predict how and where EIN2 CEND‐NLS and related EIN2‐NLS probes may bind to IMPα, we used Alphafold2 [78]. The results suggest that EIN2‐NLS may potentially bind to the major binding groove situated between ARM repeats 2–4 (major NLS binding site). However, given the amino acid (aa) conservation at this site, binding would not align with the observed differences in binding affinity of the various isoforms. In accordance with this, previous structural and calorimetric studies by de Oliveira *et al*., demonstrated that NLS peptides do not bind exclusively to the major NLS binding site [98]. Rather, they also interact with the minor NLS binding site which is located at ARM 7–8. When the minor site is considered as the EIN2‐NLS binding site, the observed differences in the experimentally determined *K*_d_ value align well with the aa substitutions observed in the various IMPα isoforms at this site. The only discrepancy was observed for IMPα6, which based on the aa composition at this site is expected to show similar affinities as IMPα1‐4.

**Fig. 9:**
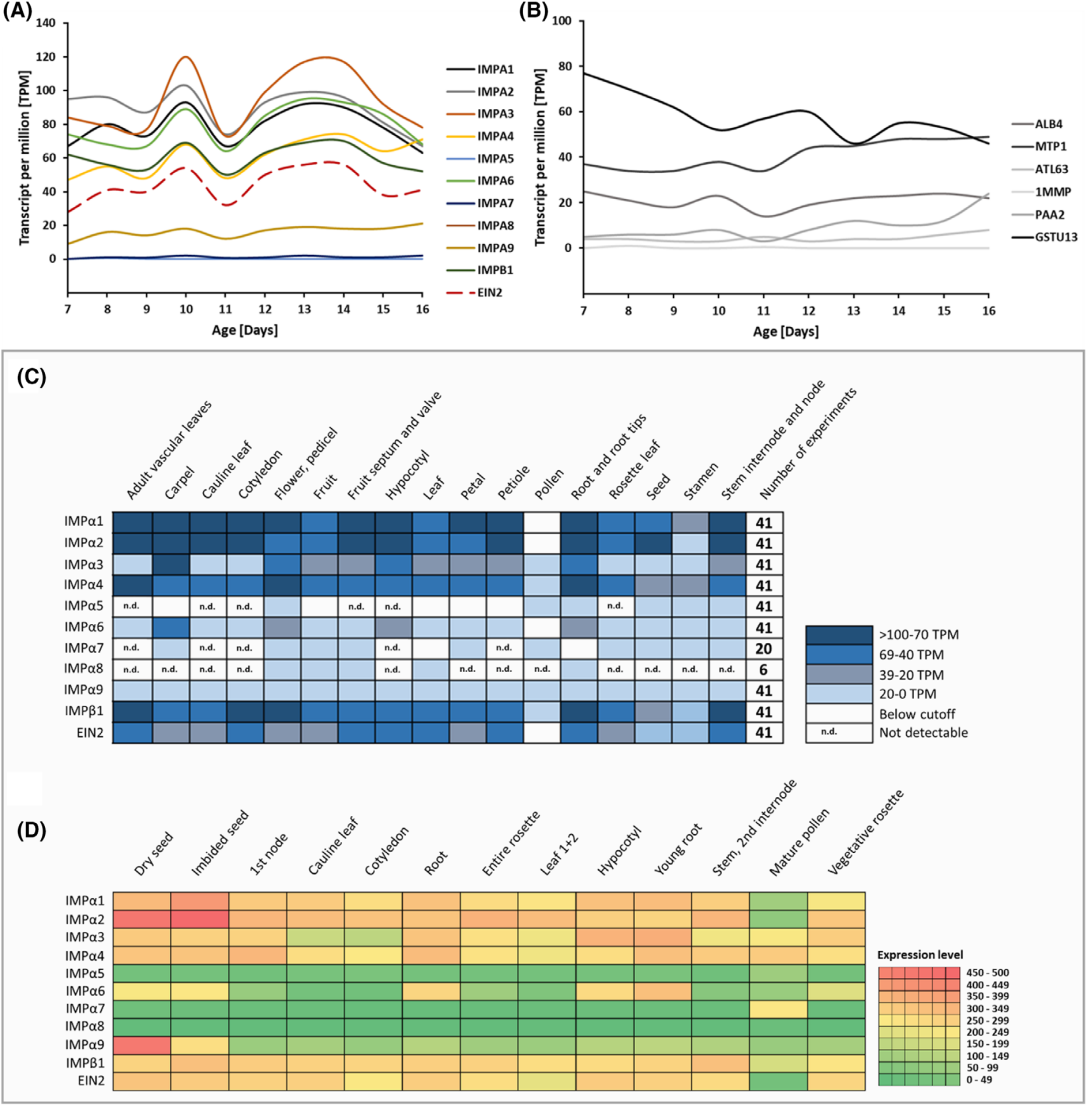
Importin and EIN2 expression levels in *A. thaliana* tissues. A and B show expression profiles of importin (IMP)α, IMPβ1, ETHYLENE INSENSITIVE 2 (EIN2), and control proteins in senescent *A. thaliana*. Based on data that are deposited at the Expression Atlas webserver [107]. Expression profiles are shown for IMPα1‐9, IMPβ1, and EIN2 (A). Expression maxima can be observed after 10 days and between 12 and 14 days. The expression of six random control proteins (*A. thaliana*) is shown (B), including ALB4 (ALBINO3‐like protein 1), MTP1 (Metal tolerance protein 1), ATL63 (RING‐H2 finger protein), 1MMP (Metalloendoproteinase), PAA2 (Copper‐transporting ATPase), and GSTU13 (Glutathione S‐transferase U13). The gray box shows the gene expression of importins and EIN2 in *A. thaliana* tissues at the RNA (C) and protein (D) levels. The results are based on data from the Expression Atlas webserver for RNA levels (IMPα1, 2, 3, 4, 6, and 9 based on 41 experiments, IMPα5 based on 12 experiments, IMPα7 based on 20, and IMPα8 based on 6 experiments) and the ePlant database for protein levels (results based on expression levels across 350 samples). The RNA levels are shown in TPM (Transcript per million), representing the number of transcripts for a given gene or isoform.

Given their high affinity, IMPα1‐4 qualifies as the preferred EIN2‐NLS interaction partner. Notably, the observed *in vitro* binding affinities are consistent with the expression pattern of the various IMPα isoforms in ethylene‐dependent developmental processes at different stages of plant life (Fig. 9).

The hormone influences early developmental aspects such as the release of seed dormancy during germination, but also late events in plant life such as flower and leaf senescence. According to the TAIR database, IMPα1‐4 show high expression during germination, in mature flowers, and senescent leaves. In contrast, low expression levels at these developmental stages are observed for IMPα5, IMPα6, IMPα8, and IMPα9, which is consistent with their lower affinity in our binding studies. Additionally, the analysis of deposited transcriptome data at the Expression Atlas database and protein expression levels at the ePlant database demonstrated that IMPαs and EIN2 show different expression levels in 17 tested *A. thaliana* tissues (Fig. 9C,D) [87, 88, 99]. Similar to the TAIR database, the strongest expression pattern could be observed for IMPα1‐4. Based on deposited data of an expression analysis in senescent *A. thaliana* [86], we observed that most of the IMPαs, IMPβ1, and EIN2 show a similar expression profile during senescence, with an expression peak after 10 days and a second broad expression maximum between 12 and 14 days (Fig. 9A). We included six control genes that show different expression profiles compared to the IMPs and EIN2 (Fig. 9B) to demonstrate the significance of the expression profile. Interestingly, IMPα7‐9 showed very low expression levels during these experiments, which may suggest that these proteins have a minor effect on the EIN2 CEND translocation. IMPα8 and 9 were excluded from the following *in planta* experiments. In contrast, the experiments were performed with IMPα7 because it showed one of the strongest *K*_d_ values against NOP‐1 with 3 ± 0.3 μm.

The TAIR database includes the expression data of *A. thaliana* IMPα1‐9 in the presence of ACC (1‐Aminocyclopropane‐1‐carboxylic acid), an ethylene precursor [100]. The experiments were conducted for 3 h, during which a significant increase in expression was only observed for IMPα5 (Fig. S4). Importins are involved in the nuclear transport of several proteins and are permanently expressed in plants. Our triple response experiments have already demonstrated that none of the nine tested IMPα isoforms in *Arabidopsis* is exclusively responsible for EIN2 CEND nucleocytoplasmic transport. Consequently, the expression level following ACC treatment—equivalent to ethylene treatment—does not increase.

The *in planta* FLIM‐FRET experiments corroborate the findings of the *in vitro* binding studies, demonstrating an interaction between EIN2^479–1294^, the cytosolic part of EIN2 that is transported into the nucleus upon ethylene sensing, and at least six out of seven tested IMPα proteins. The localization of EIN2C^479–1294^ in both the cytoplasm and the nucleus, even in the absence of co‐expression of any of the *Arabidopsis* IMPα, is most likely due to the presence of endogenous importin proteins in *N. benthamiana*, given the high degree of conservation of the IMPα‐IMPβ pathway [61, 101]. The fusion of proteins to fluorophores has been shown to alter the biophysical properties of the fluorophore [102]. Thus, it is plausible that this phenomenon may occur *vice versa*. This will result in lower affinities of the IMPα‐YFP constructs as compared to the endogenous importins, potentially due to alterations in their biophysical properties. Furthermore, the nucleocytoplasmic transport of EIN2 is a process that involves both IMPα and IMPβ proteins. Given that we only expressed IMPα proteins from *A. thaliana*, but not the related IMPβ proteins in the *N. benthamiana* leaf cells, it seems likely that the endogenous IMPα‐IMPβ interaction is stronger than the interaction between *A. thaliana* IMPα and endogenous IMPβ. According to our *in planta* studies, all IMPα isoforms other than IMPα5 bind EIN2. Further analysis revealed that this interaction is facilitated via the EIN2‐NLS motif, as no lifetime reduction was detected in samples of IMPα3 and EIN2C^479–1294^ΔNLS.

In conclusion, the results of our *in vitro* binding studies, *in planta* localization and interaction studies as well as our computational structure predictions demonstrate that EIN2‐NLS CEND can bind various members of the IMPα superfamily for mediating transport into the nucleus. Future studies aim at elucidating the precise binding site of EIN2‐NLS with the IMPα superfamily.

## Materials and methods

### Sterilization and growth of *Arabidopsis thaliana* mutants

Around 10 mg of seeds was filled into a 2‐mL reaction tube and put into a desiccator with the lid opened. Additionally, a beaker with 100 mL 37% perchloric acid was put into the desiccator. 3 mL of 37% hydrochloric acid was pipetted into the beaker to start the reaction, and the lid of the desiccator was closed immediately. The seeds were incubated for 1 h. After the incubation time, the lid of the desiccator was opened slightly, while the tubes were incubated for 20 more minutes to wait for the gas to evaporate. 1 mL of sterile water was added to the reaction tubes, and the seeds were stratified by storage at 4 °C for 3 days before planting (otherwise they can be planted directly after this procedure). Before starting to plant the seeds, any material (e.g., pots and tubs) was sterilized with 70% ethanol. A mixture of soil, vermiculite, and sand (ratio 8:1:1) was prepared, and water was added in the right proportion to make the soil mix soggy but not mushy. The soil mix was sterilized by heating it in the microwave at 800 W for about 10 min. Pots were filled with the sterilized soil mix afterwards. Around 100 μL of 1% agarose in tap water was added to the reaction tubes containing the sterilized seedlings for better pipetting precision, and the seeds were planted into the soil mix. The growth of *A. thaliana* was performed at 22 °C, 60% humidity, and 100–120 μEinstein light intensity. The plants were watered only from the bottom every 2–3 days. After 5 weeks, the plants were big enough to grow in their own pots instead of several plants being grown crowded in one pot.

### Triple response assay

The triple response media for the agar plates was prepared by mixing 0.43% (w/v) of Murashige and Skoog (MS) salts and 1% (w/v) sucrose. The pH is adjusted to 6.0 before adding 0.7% (w/v) plant agar to the media and autoclaving it at 121 °C for 30 min. Before pouring the plates, the agar media was cooled down to about 45 °C and either ACC was added to the agar for plates containing ACC or the plates were poured directly after cooling down. The plates that were used for the triple response had the dimensions of 130 × 130 × 15 mm. Sterilization of the seeds had to take place directly before planting. The seeds were applied one by one in a straight horizontal line onto the agar plates. A maximum amount of two mutants, except the wild‐type, was placed per plate. Afterwards, the plates were wrapped separately in aluminum foil and placed vertically (so that the seeds grow to the top) at 4 °C for 3 days (stratification). Subsequently, the plates were light‐treated for 2 h at room temperature, which improves germination before being covered again and placed vertically at 22 °C for 5 days. The seedlings were documented, and the length of the hypocotyl was analyzed using a binocular microscope and the software Motion 3.0, respectively.

### Transient transformation of *N. benthamiana*

A modified version of a previously described procedure was used for the transient transformation of *N. benthamiana* [17]. An overnight culture of agrobacteria was inoculated in DYT and incubated at 28°C one day before infiltration. Bacteria were then centrifuged at 4000 ***g*** and resuspended in infiltration medium (5% (w/v) Saccharose; 0.01% (w/v) MgSO_4_; 0.01% (w/v) Glucose; Acetosyringone 450 μm) to an OD600 of 0.4–0.8. Resuspended bacteria were kept on ice for 1–2 h prior to infiltration. For co‐infiltration, bacteria solutions were mixed in equal quantities, and Agrobacterium strain GV3101:p19 expressing the p19 silencing inhibitor was added to each mix. Infiltration of the bacteria solution was performed with a 1 mL syringe onto the abaxial side of 3‐ to 4‐week‐old plants. Plants were kept under continuous light before microscopy analysis. For ß‐estradiol inducible constructs, plants were sprayed on the abaxial side of infiltrated leaves with an estradiol solution (ß‐estradiol 20 μm; Tween‐20 0.1% (w/v)) prior to imaging.

### Fluorescence imaging

For fluorescence imaging, a Zeiss LSM 780 confocal microscope (40× water immersion objective, Zeiss C‐PlanApo, NA 1.2) was used. YFP was excited at 514 nm and Cerulean at 458 nm. Signal recording of each fluorophore was done within its maximum emission peak while avoiding auto‐fluorescence above 650 nm. Laser power and gain were adjusted differently for each image based on fluorophore signal intensity.

### Fluorescence lifetime imaging

Fluorescence lifetime imaging was performed using a protocol described previously [103]. Fluorescence lifetime was measured at a Zeiss LSM 780 confocal microscope (40× water immersion objective, Zeiss C‐PlanApo, NA 1.2). For TCSPC, a PicoQuant Hydra Harp 400 (PicoQuant, Berlin, Germany) was used. Photon counting was performed with a picosecond resolution. Cerulean was excited with a 440 nm (LDH‐D‐C‐485, 32 MHz, PicoQuant, Berlin, Germany) pulsed polarized laser. Laser power at the objective lens was adjusted to ~2.2 μW in *N. benthamiana* experiments. Light emitted from the sample was separated by a polarizing beam splitter before photons were selected with a band‐pass filter. A 482/35 band‐pass filter was used for Cerulean, and for YFP, a 534/30 band‐pass filter was used. Photons were detected in both donor and acceptor channels simultaneously with Tau‐SPADs (PicoQuant, Berlin, Germany). Images were acquired at zoom 8 with a resolution of 256 × 256 pixels, with a pixel size of 0.1 μm and a pixel dwell time of 12.54 μs, and a laser repetition rate of 32 MHz. Photons were collected over 40 frames. To avoid pileup effects, nuclei containing high donor concentrations were avoided. Before image acquisition, the system was calibrated for each donor. For this, the objective was adjusted to reach a maximal count rate. FCS curves of Atto425 dye and water were acquired to monitor the system function. Internal response functions for each laser were determined by measuring the fluorescence decay of quenched Atto425 in saturated KI, using the same hardware settings as for the FRET pair of interest. Fluorescence lifetime images were acquired in the cytosol, ER, or nucleus in donor‐only samples and samples with IMPα1‐4 and IMPα7. Signal intensity of IMPα5‐YFP and IMPα6‐YFP was considerably lower compared to the other IMPα‐YFP fusions, likely due to lower protein concentrations. To ensure sufficient acceptor concentrations for FRET to occur, only FLIM images of nuclei were acquired for these samples. Micrographs display representative FLIM images for each sample.

### Fitting of the fluorescence decays

The fluorescence decays were fitted using a previously reported method with minor modifications [104].

The fluorescence decays of selected ROIs in the FLIM image were analyzed with the SymPhoTime FLIM analysis software (SymPhoTime 64, version 2.4; PicoQuant, Berlin, Germany). TCSPC bins of Channels 1 and 2 (parallel and perpendicular light) were binned by 16, resulting in a bin width of 16 ps. Nuclei or cytoplasm/endoplasmic reticulum were selected by hand using the ROI tool. Chloroplasts and pixels above the pile‐up limit (10% of the laser repetition rate) were manually removed. Decays from all samples were fitted with the FLIM analysis tool (Fitting model: n‐exponential reconvolution) using two lifetimes (model parameter *n* = 2). FRET efficiency was calculated as the lifetime of the FRET sample over the arithmetic mean of the lifetimes of the donor‐only samples measured on the same day.

### Expression of importin alpha proteins and GB1‐ *At*EIN2C^1215^ ^−1281^

Genes encoding several isoforms of the IMPα superfamily were either isolated from *Arabidopsis thaliana* (IMPα1, ‐2, ‐3, ‐4, ‐8) or synthetically produced by GenScript (IMPα5, ‐6, ‐7, ‐9) and cloned into the pETEV21a vector (modified version of the pET21a vector with an additional TEV cleavage site between the GOI and the His‐tag), which contains a C‐terminal hexa‐His‐tag. Only IMPα5 was additionally cloned into the pETEV16b vector (modified version of the pET16b vector with an additional TEV cleavage site between the GOI and the His‐tag), which contains an N‐terminal deca‐His‐tag (see Table S2 for primer list). The *At*EIN2C^1215–1281^ gene was cloned into the pETEV16b vector and additionally contained a GB1‐domain at the N terminus to enhance solubility. Most of the proteins were expressed in the *E. coli* BL21pRARE strain, while IMPα7, IMPα8, and ΔIBB_IMPα1 (lack of the IBB domain) were expressed using the *E. coli* BL21gold strain. The bacterial cultures were adjusted to an initial OD_600_ of 0.1 and grown in 2YT media at 37 °C until an OD_600_ between 0.6 and 0.8 was reached. Subsequently, the protein expression was induced with 0.5 mm IPTG. After two hours, the cells were harvested via centrifugation at 10,000 ***g*** for 10 min. IMPα5, IMPα6, IMPα7, IMPα8, ΔIBB_IMPα1, and GB1_*At*EIN2C^1215–1281^ were expressed at 16 °C overnight with the addition of 2% (v/v) ethanol. The cell pellets were frozen in liquid nitrogen and stored at −20 °C.

### Protein purification

The stored cell pellet was resuspended in 1× PBS, 5% (w/v) Glycerol and 0.1% (v/v) NP40 pH 7.4 (5 mL per g cell). A spatula tip of DNase and 4–6 drops of Antifoam were added to the resuspended cells, which were disrupted by using the cell disruptor afterwards. After cell disruption, cell debris and insoluble protein were separated by centrifugation at 100,000 ***g*** for one hour, and subsequently, the supernatant was loaded onto a pre‐equilibrated Ni^2+^‐IMAC (immobilized metal affinity chromatography) column. The column was washed with 500 mm NaCl and 50 mm NaP_i_ buffer pH 7.4 until the baseline was reached again. Afterwards, unspecific proteins were washed off stepwise using 500 mm NaCl and 50 mm NaP_i_ buffer with increasing imidazole concentrations (50 mm and 100 mm imidazole), while the proteins of interest were eluted with 500 mm NaCl, 50 mm NaP_i_ buffer and 500 mm imidazole pH 7.4. The purified proteins were concentrated using ultrafiltration units (Amicon), frozen with liquid nitrogen, and stored at −80 °C.

### Peptides

All NLS peptides were purchased from GenScript (NJ, USA) as a lyophilized powder with >95% purity. NOP‐1 consists of 8 amino acid residues and N41P‐1 consists of 41 amino acid residues. The C‐termini of the d‐ri‐peptides are amidated, and the N‐termini are acetylated. An analysis certificate from GenScript demonstrated the purity of each peptide (for details see Figs S5–S8).

### Synthesis of fluorescently labeled NOP‐1

The TAMRA‐NOP‐1 peptide was synthesized using solid‐phase peptide synthesis (SPPS) with the Activotec P11 automated synthesizer (Activotec, Cambridge, UK) following the standard fluorenylmethoxycarbonyl (Fmoc) protocol. Depending on the NOP‐1 sequence (LKRYKRRL), the following amino acids were used: Fmoc‐Lys(Boc)‐OH (99.5%), Fmoc‐Tyr(tBu)‐OH (98%) (BLD Pharmatech GmbH, Reinbek, Germany), Fmoc‐Arg(Pbf)‐OH (98%), and Fmoc‐Leu‐OH (98%) (Carbolution GmbH, Sankt Ingbert, Germany). Fmoc‐protected amino acids were employed throughout the synthesis. The solid phase consisted of H‐Leu‐2‐CT‐polystyrene resin (loading 1.13 mmol·g^−1^) (RAPP Polymere GmbH, Tübingen, Germany). The synthesis consisted of repetitive Fmoc deprotection and amino acid coupling steps. Fmoc cleavage was carried out by treating the resin three times with 25% (v/v) piperidine (Thermo Fisher Scientific) in dimethylformamide (DMF) (Biosolve, Valkenswaard, the Netherlands) for 10, 15, and 20 min, respectively. After each treatment, the resin was washed 10 times with DMF. Amino acid coupling was performed by treating the resin with a solution containing 5 eq. of Fmoc‐amino acid, 5 eq. of benzotriazol‐1‐yloxytripyrrolidinophosphonium hexafluoro‐phosphate (PyBOP) (Biosynth, Staad, Switzerland), and 10 eq. of diisopropylethylamine (DIPEA) (Carl Roth) in 2 mL of DMF for 1 h. After the coupling reaction, the resin was washed 10 times with dichloromethane (DCM) (Merck, Darmstadt, Germany) and DMF. For fluorophore conjugation, 1.3 eq. of 5‐Carboxytetramethylrhodamine succinimidyl ester (TAMRA‐NHS) (Biosynth, Staad, Switzerland) ester was dissolved in 4 mL of DMF, and 10 eq. of DIPEA were added to the solution. This mixture was incubated with the resin‐bound peptide for 16 h in the absence of light. After conjugation, the resin was washed 10 times with DMF, followed by alternating washes with DCM and methanol until the washing solution became clear. Peptide cleavage from the resin was achieved by treatment with 2 mL of 70% (v/v) trifluoroacetic acid (TFA) (Fisher Chemical, Schwerte, Germany) and 30% (v/v) triisopropylsilane (TIPS) (Fluorochem, Hadfield, UK) for 1 h. The cleaved peptide was precipitated with diethyl ether (honey well). To remove TFA salts, an anionic exchange was performed using AG®1‐X8 resin (Bio Rad, Hercules, USA). The resin was activated by sequential washes with 1.6 N acetic acid and 0.16 N acetic acid (Merck, Darmstadt, Germany). The peptide solution was incubated with the activated resin for 1 h and then lyophilized to yield the purified peptide.

### Reversed phase‐ high pressure liquid chromatography (RP‐HPLC) electron spray ionization‐ mass spectrometry (ESI‐MS)

RP‐HPLC‐MS spectra were performed on an Agilent Technologies 1260 Infinity Instrument in combination with a 6120‐quadrupole mass spectrometer (Figs S9, S10). The instrument has a wavelength detector (VWD1 A) that measures the absorbance at 214 nm. The mass spectrometer generates ions using the electrospray method, which is used at a Charge‐to‐mass ratio detected between 200 and 2000. The separation of the sample was performed on the MZ‐AquaPerfect C18 column (3.0 × 50 mm, 3 μm) at 25 °C. As mobile Phase the following mixtures were used: (A) H_2_O/acetonitrile (95:5 Vol.%) with 0.1 Vol.% Formic acid and (B) H_2_O/acetonitrile (5:95 Vol.%) with 0.1 Vol.% formic acid. The flow rate was 0.4 mL·min_−2_. A linear gradient from 100% A/0% B to 0% A/100% B with a total measurement time of 17 min.

### Biolayer interferometry (BLI) measurements

An Octet K2 device (Fortè Bio, Dallas, USA) was used to obtain BLI binding data, which were processed using the system's built‐in software. Experiments were conducted at room temperature with continuous shaking at 400 rpm in black 96‐well plates, each well containing 200 μL. Biotinylated ΔIBB_IMPα1 and IMPα4 were loaded onto a streptavidin (SA) biosensor (Fortè Bio) at a concentration of 15 μm in 100 mm Tris (pH 7.0), 100 mm KCl, 0.01% (w/v) Tween 20 for 600 s. Therefore, ΔIBB_IMPα1 and IMPα4 were biotinylated using the Biotin‐PEG_4_‐NHS ester Protein Labeling Kit from Jena Bioscience (Jena, Germany). Evaluation of successful biotinylation was performed with the Colorimetric Biotin Assay Kit from Sigma (St. Louis, USA). After establishing a baseline in the binding buffer, biosensors were dipped into peptide‐containing wells at the indicated concentrations for 300 s to monitor association, followed by a 300 s dissociation phase in binding buffer. For reliable dissociation constant (*K*_d_) measurements, BLI experiments were conducted with a range of peptide and protein concentrations tailored to their binding strengths. Stock solutions of peptides and proteins were diluted in binding buffer. The resulting raw data were corrected for background using reference sensors exposed only to buffer. All measurements were performed in quadruplicate. The raw data were evaluated with the octet data analysis software. Curve fitting was completed with ForteBio Biosystems using a heterogenous fitting algorithm and 1:2 binding model to obtain *K*_d_ values.

### Fluorescence polarization (FP) measurements

The FP measurements were performed using the LS55 Fluorescence Spectrometer from PerkinElmer and a precision cuvette consisting of SUPRASIL® from Hellma. TAMRA‐NOP‐1 was used at a concentration of 250 nm and a detector voltage of 790 V, whereas Alexa488_GB1_*At*EIN2C^1215–1281^ was used at a concentration of 200 nm and a detector voltage of 770 V. Samples were prepared in a total volume of 50 μL with constant fluorophore but varying protein concentrations. The measurement was tracked for 100 s, and the polarization value was determined by averaging half of the recorded time period. The competition experiments were performed analogously with the exception that both the fluorophore and protein concentration were held constant, while the competitor concentration was varied. The protein concentration used in the competition experiments was identical to the determined *K*_d_ value resulting from the non‐competition experiments.

### *In silico* analysis

Importin α superfamily protein sequences were retrieved from the NCBI database, and sequence alignments were performed using T‐COFFEE [79]. The I‐TASSER server was used to construct 3‐dimensional homology models of IMPα4 and ΔIBB_IMPα1 [105]. The IMPα homology models were calculated based on 10 proteins with highly similar structures in PDB, as identified by the TM‐alignment algorithm. TM‐align is a computer algorithm for protein structure alignment using dynamic programming and the TM‐score rotation matrix. An optimal alignment between two proteins, as well as the TM‐score, will be reported for each comparison. In general, a TM‐score >0.5 means the structures share the same fold [105]. For all 10 structures used for the ΔIBB_IMPα1 model (PDB codes: 2YNS, 4RXH, 1WA5, 4UAE, 1IAL, 2JDQ, 4RV1, 5XJG, 4R0Z, and 7V7B), the TM‐score shows values >0.75. For all 10 structures used for the IMPα4 model (PDB codes: 1WA5, 2YNS, 4RXH, 1IAL, 5XJG, 4UAE, 2JDQ, 4RV1, 5HB4, and 4R0Z), the TM‐score shows values >0.65. The model of *A. thaliana* IMPα1‐IMPβ1‐NOP‐1 was generated using the AlphaFold 3 web server [106]. All figures are made using the PyMOL Molecular Graphics System, Version 1.3, Schrödinger, LLC.

### Statistical analysis

For statistical analysis, GraphPad Prism 6 was used. For the triple response assay and the *in planta* FLIM‐FRET experiments, two‐way ANOVA, Kruskal–Wallis test, Dunnett's multiple comparisons test, and t‐test were performed.

## Conflict of interest

The authors declare that they have no known competing financial interests or personal relationships that could have appeared to influence the work reported in this paper.

## Author contributions

Conceptualization, GG; Methodology, FW, GG, RJE; Investigation, FW, JEM, RJE, NJ, MW, and LH; Formal analysis, FW, JEM, RJE, NJ, MW, and LH; Validation, FW, JEM, RJE, NJ, RS and GG; Visualization, FW and RJE; Writing – Original draft, FW, JEM, RJE, and GG; Writing – Review and Editing, FW, RJE, MW, LH, RS, and GG. All authors read and edited the manuscript prior to publication.

## Supporting information


**Fig. S1.** Sequence alignment of *A. thaliana* IMPα1‐9.
**Fig. S2.** Alignment of amino acid sequences of the nine IMPα isoforms at the minor binding site.
**Fig. S3.** Alphafold model of the IMPα1‐IMPβ1‐NOP‐1 complex.
**Fig. S4.** IMPα protein family expression levels in *A. thaliana* after ACC treatment.
**Fig. S5.** HPLC chromatogram of NOP‐1.
**Fig. S6.** Mass spectrometry of NOP‐1.
**Fig. S7.** HPLC chromatogram of N41P‐1.
**Fig. S8.** Mass spectrometry of N41P‐1.
**Fig. S9.** Reversed phase (RP) HPLC chromatogram of TAMRA‐NOP‐1.
**Fig. S10.** Mass spectrometry analysis of TAMRA‐NOP‐1.
**Table S1.** EIN2‐NLS peptide/protein sequences investigated in this study.
**Table S2.** List of primers used for PCR amplification and cloning of IMPα genes.

## Data Availability

All relevant data are included in this manuscript and its supporting information. Further inquiries or requests for materials can be directed to georg.groth@hhu.de.

## References

[1] Abeles FB , Morgan PW & Saltveit ME (1992) Ethylene in Plant Biology. Academic Press, San Diego, CA.

[2] Ecker JR (1995) The ethylene signal transduction pathway in plants. Science 268, 667–675. 7732375 10.1126/science.7732375

[3] Ju C , Van de Poel B , Cooper ED , Thierer JH , Gibbons TR , Delwiche CF & Chang C (2015) Conservation of ethylene as a plant hormone over 450 million years of evolution. Nat Plants 1, 1–7. 10.1038/nplants.2014.4 27246051

[4] Hamilton A , Lycett G & Grierson D (1990) Antisense gene that inhibits synthesis of the hormone ethylene in transgenic plants. Nature 346, 284–287.

[5] Wilkinson JQ , Lanahan MB , Yen H‐C , Giovannoni JJ & Klee HJ (1995) An ethylene‐inducible component of signal transduction encoded by never‐ripe. Science 270, 1807–1809. 8525371 10.1126/science.270.5243.1807

[6] Chen YF , Gao Z , Kerris RJ 3rd , Wang W , Binder BM & Schaller GE (2010) Ethylene receptors function as components of high‐molecular‐mass protein complexes in Arabidopsis. PLoS One 5, e8640. 20062808 10.1371/journal.pone.0008640 PMC2799528

[7] Grefen C , Stadele K , Ruzicka K , Obrdlik P , Harter K & Horak J (2008) Subcellular localization and in vivo interactions of the *Arabidopsis thaliana* ethylene receptor family members. Mol Plant 1, 308–320. 19825542 10.1093/mp/ssm015

[8] Dong CH , Rivarola M , Resnick JS , Maggin BD & Chang C (2008) Subcellular co‐localization of Arabidopsis RTE1 and ETR1 supports a regulatory role for RTE1 in ETR1 ethylene signaling. Plant J 53, 275–286. 17999643 10.1111/j.1365-313X.2007.03339.x PMC2194639

[9] Bleecker AB , Estelle MA , Somerville C & Kende H (1988) Insensitivity to ethylene conferred by a dominant mutation in *Arabidopsis thaliana* . Science 241, 1086–1089. 17747490 10.1126/science.241.4869.1086

[10] Chang C , Kwok SF , Bleecker AB & Meyerowitz EM (1993) Arabidopsis ethylene‐response gene ETR1: similarity of product to two‐component regulators. Science 262, 539–544. 8211181 10.1126/science.8211181

[11] Hua J , Chang C , Sun Q & Meyerowitz EM (1995) Ethylene insensitivity conferred by Arabidopsis ERS gene. Science 269, 1712–1714. 7569898 10.1126/science.7569898

[12] Hua J , Sakai H , Nourizadeh S , Chen QG , Bleecker AB , Ecker JR & Meyerowitz EM (1998) EIN4 and ERS2 are members of the putative ethylene receptor gene family in Arabidopsis. Plant Cell 10, 1321–1332. 9707532 10.1105/tpc.10.8.1321 PMC144061

[13] Hua J & Meyerowitz EM (1998) Ethylene responses are negatively regulated by a receptor gene family in *Arabidopsis thaliana* . Cell 94, 261–271. 9695954 10.1016/s0092-8674(00)81425-7

[14] Guo H & Ecker JR (2004) The ethylene signaling pathway: new insights. Curr Opin Plant Biol 7, 40–49. 14732440 10.1016/j.pbi.2003.11.011

[15] Kieber JJ , Rothenberg M , Roman G , Feldmann KA & Ecker JR (1993) CTR1, a negative regulator of the ethylene response pathway in Arabidopsis, encodes a member of the raf family of protein kinases. Cell 72, 427–441. 8431946 10.1016/0092-8674(93)90119-b

[16] Alonso JM , Hirayama T , Roman G , Nourizadeh S & Ecker JR (1999) EIN2, a bifunctional transducer of ethylene and stress responses in Arabidopsis. Science 284, 2148–2152. 10381874 10.1126/science.284.5423.2148

[17] Bisson MM , Bleckmann A , Allekotte S & Groth G (2009) EIN2, the central regulator of ethylene signalling, is localized at the ER membrane where it interacts with the ethylene receptor ETR1. Biochem J 424, 1–6. 19769567 10.1042/BJ20091102

[18] Bisson MM & Groth G (2010) New insight in ethylene signaling: autokinase activity of ETR1 modulates the interaction of receptors and EIN2. Mol Plant 3, 882–889. 20591837 10.1093/mp/ssq036

[19] Huang Y , Li H , Hutchison CE , Laskey J & Kieber JJ (2003) Biochemical and functional analysis of CTR1, a protein kinase that negatively regulates ethylene signaling in Arabidopsis. Plant J 33, 221–233. 12535337 10.1046/j.1365-313x.2003.01620.x

[20] Bisson MM & Groth G (2011) New paradigm in ethylene signaling: EIN2, the central regulator of the signaling pathway, interacts directly with the upstream receptors. Plant Signaling & Behavior 6, 164–166. 21242723 10.4161/psb.6.1.14034 PMC3122035

[21] Merchante C , Brumos J , Yun J , Hu Q , Spencer KR , Enríquez P , Binder BM , Heber S , Stepanova AN & Alonso JM (2015) Gene‐specific translation regulation mediated by the hormone‐signaling molecule EIN2. Cell 163, 684–697. 26496608 10.1016/j.cell.2015.09.036

[22] Li W , Ma M , Feng Y , Li H , Wang Y , Ma Y , Li M , An F & Guo H (2015) EIN2‐directed translational regulation of ethylene signaling in Arabidopsis. Cell 163, 670–683. 26496607 10.1016/j.cell.2015.09.037

[23] Johnson PR & Ecker JR (1998) The ethylene gas signal transduction pathway: a molecular perspective. Annu Rev Genet 32, 227–254. 9928480 10.1146/annurev.genet.32.1.227

[24] Wang F , Cui X , Sun Y & Dong C‐H (2013) Ethylene signaling and regulation in plant growth and stress responses. Plant Cell Rep 32, 1099–1109. 23525746 10.1007/s00299-013-1421-6

[25] Bisson MM & Groth G (2015) Targeting plant ethylene responses by controlling essential protein‐protein interactions in the ethylene pathway. Mol Plant 8, 1165–1174. 25843012 10.1016/j.molp.2015.03.014

[26] Milic D , Dick M , Mulnaes D , Pfleger C , Kinnen A , Gohlke H & Groth G (2018) Recognition motif and mechanism of ripening inhibitory peptides in plant hormone receptor ETR1. Sci Rep 8, 3890. 29497085 10.1038/s41598-018-21952-3 PMC5832771

[27] Bisson MM , Kessenbrock M , Muller L , Hofmann A , Schmitz F , Cristescu SM & Groth G (2016) Peptides interfering with protein‐protein interactions in the ethylene signaling pathway delay tomato fruit ripening. Sci Rep 6, 30634. 27477591 10.1038/srep30634 PMC4967898

[28] Klein S , Fiebig A , Neuwald D , Dluhosch D , Muller L , Groth G , Noga G & Hunsche M (2019) Influence of the ethylene‐related signal‐inhibiting octapeptide NOP‐1 on postharvest ripening and quality of ‘Golden delicious’ apples. J Sci Food Agric 99, 3903–3909. 30693519 10.1002/jsfa.9613

[29] Kessenbrock M , Klein SM , Muller L , Hunsche M , Noga G & Groth G (2017) Novel protein‐protein inhibitor based approach to control plant ethylene responses: synthetic peptides for ripening control. Front Plant Sci 8, 1528. 28928762 10.3389/fpls.2017.01528 PMC5591945

[30] Hoppen C , Muller L , Albrecht AC & Groth G (2019) The NOP‐1 peptide derived from the central regulator of ethylene signaling EIN2 delays floral senescence in cut flowers. Sci Rep 9, 1287. 30718569 10.1038/s41598-018-37571-x PMC6361973

[31] Qiao H , Shen Z , Huang S‐s C , Schmitz RJ , Urich MA , Briggs SP & Ecker JR (2012) Processing and subcellular trafficking of ER‐tethered EIN2 control response to ethylene gas. Science 338, 390–393. 22936567 10.1126/science.1225974 PMC3523706

[32] Wen X , Zhang C , Ji Y , Zhao Q , He W , An F , Jiang L & Guo H (2012) Activation of ethylene signaling is mediated by nuclear translocation of the cleaved EIN2 carboxyl terminus. Cell Res 22, 1613–1616. 23070300 10.1038/cr.2012.145 PMC3494400

[33] Ju C , Yoon GM , Shemansky JM , Lin DY , Ying ZI , Chang J , Garrett WM , Kessenbrock M , Groth G , Tucker ML *et al*. (2012) CTR1 phosphorylates the central regulator EIN2 to control ethylene hormone signaling from the ER membrane to the nucleus in Arabidopsis. Proc Natl Acad Sci U S A 109, 19486–19491. 23132950 10.1073/pnas.1214848109 PMC3511113

[34] Zhang F , Qi B , Wang L , Zhao B , Rode S , Riggan ND , Ecker JR & Qiao H (2016) EIN2‐dependent regulation of acetylation of histone H3K14 and non‐canonical histone H3K23 in ethylene signalling. Nat Commun 7, 13018. 27694846 10.1038/ncomms13018 PMC5063967

[35] Chao Q , Rothenberg M , Solano R , Roman G , Terzaghi W & Ecker JR (1997) Activation of the ethylene gas response pathway in Arabidopsis by the nuclear protein ETHYLENE‐INSENSITIVE3 and related proteins. Cell 89, 1133–1144. 9215635 10.1016/s0092-8674(00)80300-1

[36] Solano R , Stepanova A , Chao Q & Ecker JR (1998) Nuclear events in ethylene signaling: a transcriptional cascade mediated by ETHYLENE‐INSENSITIVE3 and ETHYLENE‐RESPONSE‐FACTOR1. Genes Dev 12, 3703–3714. 9851977 10.1101/gad.12.23.3703 PMC317251

[37] Li Q , Fu H , Yu X , Wen X , Guo H , Guo Y & Li J (2024) The SALT OVERLY SENSITIVE 2–CONSTITUTIVE TRIPLE RESPONSE1 module coordinates plant growth and salt tolerance in Arabidopsis. J Exp Bot 75, 391–404. 37721807 10.1093/jxb/erad368

[38] Zhang J , Chen Y , Lu J , Zhang Y & Wen C‐K (2020) Uncertainty of EIN2Ser645/Ser924 inactivation by CTR1‐mediated phosphorylation reveals the complexity of ethylene signaling. Plant Communications 1, 100046. 33367241 10.1016/j.xplc.2020.100046 PMC7747984

[39] Frey S , Richter RP & Görlich D (2006) FG‐rich repeats of nuclear pore proteins form a three‐dimensional meshwork with hydrogel‐like properties. Science 314, 815–817. 17082456 10.1126/science.1132516

[40] Lüdke D , Rohmann PF & Wiermer M (2021) Nucleocytoplasmic communication in healthy and diseased plant tissues. Front Plant Sci 12, 719453. 34394173 10.3389/fpls.2021.719453 PMC8357054

[41] Frey S & Görlich D (2009) FG/FxFG as well as GLFG repeats form a selective permeability barrier with self‐healing properties. EMBO J 28, 2554–2567. 19680227 10.1038/emboj.2009.199 PMC2728434

[42] Stewart M (2007) Molecular mechanism of the nuclear protein import cycle. Nat Rev Mol Cell Biol 8, 195–208. 17287812 10.1038/nrm2114

[43] Tran EJ , Bolger TA & Wente SR (2007) SnapShot: nuclear transport. Cell 131, 420. 17956740 10.1016/j.cell.2007.10.015

[44] Tang Y , Yang X , Huang A , Seong K , Ye M , Li M , Zhao Q , Krasileva K & Gu Y (2024) Proxiome assembly of the plant nuclear pore reveals an essential hub for gene expression regulation. Nat Plants 10, 1–13. 38773271 10.1038/s41477-024-01698-9

[45] Chang C‐W , Couñago RM , Williams SJ , Boden M & Kobe B (2013) The distribution of different classes of nuclear localization signals (NLSs) in diverse organisms and the utilization of the minor NLS‐binding site inplantnuclear import factor importin‐α. Plant Signal Behav 8, 5074–5088. 10.4161/psb.25976 PMC4091121 24270630

[46] Corbett AH & Silver PA (1997) Nucleocytoplasmic transport of macromolecules. Microbiol Mol Biol Rev 61, 193–211. 9184010 10.1128/mmbr.61.2.193-211.1997 PMC232607

[47] Nigg EA (1997) Nucleocytoplasmic transport: signals, mechanisms and regulation. Nature 386, 779–787. 9126736 10.1038/386779a0

[48] Fried H & Kutay U (2003) Nucleocytoplasmic transport: taking an inventory. Cell Molec Life Sci 60, 1659–1688. 14504656 10.1007/s00018-003-3070-3 PMC11138860

[49] Yang Y , Guo L , Chen L , Gong B , Jia D & Sun Q (2023) Nuclear transport proteins: structure, function, and disease relevance. Signal Transduct Target Ther 8, 425. 37945593 10.1038/s41392-023-01649-4 PMC10636164

[50] Görlich D , Vogel F , Mills AD , Hartmann E & Laskey RA (1995) Distinct functions for the two importin subunits in nuclear protein import. Nature 377, 246–248. 7675110 10.1038/377246a0

[51] Radu A , Blobel G & Moore MS (1995) Identification of a protein complex that is required for nuclear protein import and mediates docking of import substrate to distinct nucleoporins. Proc Natl Acad Sci 92, 1769–1773. 7878057 10.1073/pnas.92.5.1769 PMC42601

[52] Izaurralde E , Kutay U , Von Kobbe C , Mattaj IW & Görlich D (1997) The asymmetric distribution of the constituents of the ran system is essential for transport into and out of the nucleus. EMBO J 16, 6535–6547. 9351834 10.1093/emboj/16.21.6535 PMC1170258

[53] Nielsen E (2020) The small GTPase superfamily in plants: a conserved regulatory module with novel functions. Annu Rev Plant Biol 71, 247–272. 32442390 10.1146/annurev-arplant-112619-025827

[54] Terry LJ , Shows EB & Wente SR (2007) Crossing the nuclear envelope: hierarchical regulation of nucleocytoplasmic transport. Science 318, 1412–1416. 18048681 10.1126/science.1142204

[55] Görlich D , Panté N , Kutay U , Aebi U & Bischoff FR (1996) Identification of different roles for RanGDP and RanGTP in nuclear protein import. EMBO J 15, 5584–5594. 8896452 PMC452303

[56] Moroianu J , Blobel G & Radu A (1996) Nuclear protein import: ran‐GTP dissociates the karyopherin alphabeta heterodimer by displacing alpha from an overlapping binding site on beta. Proc Natl Acad Sci 93, 7059–7062. 8692944 10.1073/pnas.93.14.7059 PMC38935

[57] Christie M , Chang C‐W , Róna G , Smith KM , Stewart AG , Takeda AA , Fontes MR , Stewart M , Vértessy BG & Forwood JK (2016) Structural biology and regulation of protein import into the nucleus. J Mol Biol 428, 2060–2090. 26523678 10.1016/j.jmb.2015.10.023

[58] Görlich D & Kutay U (1999) Transport between the cell nucleus and the cytoplasm. Annu Rev Cell Dev Biol 15, 607–660. 10611974 10.1146/annurev.cellbio.15.1.607

[59] Matsuura Y , Lange A , Harreman MT , Corbett AH & Stewart M (2003) Structural basis for Nup2p function in cargo release and karyopherin recycling in nuclear import. EMBO J 22, 5358–5369. 14532109 10.1093/emboj/cdg538 PMC213792

[60] Lu K , Zhang L , Qin L , Chen X , Wang X , Zhang M & Dong H (2023) Importin β1 mediates nuclear entry of EIN2C to confer the phloem‐based defense against aphids. Int J Mol Sci 24, 8545. 37239892 10.3390/ijms24108545 PMC10217940

[61] Goldfarb DS , Corbett AH , Mason DA , Harreman MT & Adam SA (2004) Importin α: a multipurpose nuclear‐transport receptor. Trends Cell Biol 14, 505–514. 15350979 10.1016/j.tcb.2004.07.016

[62] Marfori M , Mynott A , Ellis JJ , Mehdi AM , Saunders NF , Curmi PM , Forwood JK , Bodén M & Kobe B (2011) Molecular basis for specificity of nuclear import and prediction of nuclear localization. Biochim Biophys Acta 1813, 1562–1577. 20977914 10.1016/j.bbamcr.2010.10.013

[63] Conti E , Uy M , Leighton L , Blobel G & Kuriyan J (1998) Crystallographic analysis of the recognition of a nuclear localization signal by the nuclear import factor karyopherin alpha. Cell 94, 193–204. 9695948 10.1016/s0092-8674(00)81419-1

[64] Dias SM , Wilson KF , Rojas KS , Ambrosio AL & Cerione RA (2009) The molecular basis for the regulation of the cap‐binding complex by the importins. Nat Struct Mol Biol 16, 930–937. 19668212 10.1038/nsmb.1649 PMC2782468

[65] Chang C‐W , Couñago RLM , Williams SJ , Bodén M & Kobe B (2012) Crystal structure of rice importin‐α and structural basis of its interaction with plant‐specific nuclear localization signals. Plant Cell 24, 5074–5088. 23250448 10.1105/tpc.112.104422 PMC3556976

[66] Cingolani G , Petosa C , Weis K & Müller CW (1999) Structure of importin‐β bound to the IBB domain of importin‐α. Nature 399, 221–229. 10353244 10.1038/20367

[67] Roman N , Christie M , Swarbrick CM , Kobe B & Forwood JK (2013) Structural characterisation of the nuclear import receptor importin alpha in complex with the bipartite NLS of Prp20. PLoS One 8, e82038. 24339986 10.1371/journal.pone.0082038 PMC3858281

[68] Kobe B (1999) Autoinhibition by an internal nuclear localization signal revealed by the crystal structure of mammalian importin α. Nat Struct Biol 6, 388–397. 10201409 10.1038/7625

[69] Vetter IR , Arndt A , Kutay U , Görlich D & Wittinghofer A (1999) Structural view of the ran–importin β interaction at 2.3 Å resolution. Cell 97, 635–646. 10367892 10.1016/s0092-8674(00)80774-6

[70] Tamura K & Hara‐Nishimura I (2014) Functional insights of nucleocytoplasmic transport in plants. Front Plant Sci 5, 118. 24765097 10.3389/fpls.2014.00118 PMC3980095

[71] Wang W , Ye R , Xin Y , Fang X , Li C , Shi H , Zhou X & Qi Y (2011) An importin β protein negatively regulates microRNA activity in Arabidopsis. Plant Cell 23, 3565–3576. 21984696 10.1105/tpc.111.091058 PMC3229135

[72] Guzman P & Ecker JR (1990) Exploiting the triple response of Arabidopsis to identify ethylene‐related mutants. Plant Cell 2, 513–523. 2152173 10.1105/tpc.2.6.513 PMC159907

[73] Van Der Straeten D , Djudzman A , Van Caeneghem W , Smalle J & Van Montagu M (1993) Genetic and physiological analysis of a new locus in Arabidopsis that confers resistance to 1‐aminocyclopropane‐1‐carboxylic acid and ethylene and specifically affects the ethylene signal transduction pathway. Plant Physiol 102, 401–408. 12231830 10.1104/pp.102.2.401 PMC158793

[74] Lüdke D , Roth C , Kamrad SA , Messerschmidt J , Hartken D , Appel J , Hörnich BF , Yan Q , Kusch S & Klenke M (2021) Functional requirement of the Arabidopsis importin‐α nuclear transport receptor family in autoimmunity mediated by the NLR protein SNC1. Plant J 105, 994–1009. 33210758 10.1111/tpj.15082

[75] Moerke NJ (2009) Fluorescence polarization (FP) assays for monitoring peptide‐protein or nucleic acid‐protein binding. Curr Protocols Chem Biol 1, 1–15. 10.1002/9780470559277.ch090102 23839960

[76] Wirthmueller L , Roth C , Fabro G , Caillaud MC , Rallapalli G , Asai S , Sklenar J , Jones AM , Wiermer M & Jones JD (2015) Probing formation of cargo/importin‐α transport complexes in plant cells using a pathogen effector. Plant J 81, 40–52. 25284001 10.1111/tpj.12691 PMC4350430

[77] Jug A , Bratkovič T & Ilaš J (2024) Biolayer interferometry and its applications in drug discovery and development. TrAC Trends Anal Chem 176, 117741.

[78] Jumper J , Evans R , Pritzel A , Green T , Figurnov M , Ronneberger O , Tunyasuvunakool K , Bates R , Žídek A & Potapenko A (2021) Highly accurate protein structure prediction with AlphaFold. Nature 596, 583–589. 34265844 10.1038/s41586-021-03819-2 PMC8371605

[79] Notredame C , Higgins DG & Heringa J (2000) T‐Coffee: a novel method for fast and accurate multiple sequence alignment. J Mol Biol 302, 205–217. 10964570 10.1006/jmbi.2000.4042

[80] Fanara P , Hodel MR , Corbett AH & Hodel AE (2000) Quantitative analysis of nuclear localization signal (NLS)‐importin α interaction through fluorescence depolarization: evidence for auto‐inhibitory regulation of NLS binding. J Biol Chem 275, 21218–21223. 10806202 10.1074/jbc.M002217200

[81] Harreman MT , Cohen PE , Hodel MR , Truscott GJ , Corbett AH & Hodel AE (2003) Characterization of the auto‐inhibitory sequence within the N‐terminal domain of importin α. J Biol Chem 278, 21361–21369. 12672802 10.1074/jbc.M301114200

[82] Kosugi S , Hasebe M , Matsumura N , Takashima H , Miyamoto‐Sato E , Tomita M & Yanagawa H (2009) Six classes of nuclear localization signals specific to different binding grooves of importin α. J Biol Chem 284, 478–485. 19001369 10.1074/jbc.M807017200

[83] Pang X & Zhou H‐X (2014) Design rules for selective binding of nuclear localization signals to minor site of importin α. PLoS One 9, e91025. 24609064 10.1371/journal.pone.0091025 PMC3946659

[84] Park MY , Wu G , Gonzalez‐Sulser A , Vaucheret H & Poethig RS (2005) Nuclear processing and export of microRNAs in Arabidopsis. Proc Natl Acad Sci 102, 3691–3696. 15738428 10.1073/pnas.0405570102 PMC553294

[85] Dey V & Patankar S (2018) Molecular basis for the lack of auto‐inhibition of plasmodium falciparum importin α. Biochem Biophys Res Commun 503, 1792–1797. 30064907 10.1016/j.bbrc.2018.07.115

[86] Klepikova AV , Logacheva MD , Dmitriev SE & Penin AA (2015) RNA‐seq analysis of an apical meristem time series reveals a critical point in *Arabidopsis thaliana* flower initiation. BMC Genomics 16, 1–16. 26084880 10.1186/s12864-015-1688-9 PMC4470339

[87] Mergner J , Frejno M , List M , Papacek M , Chen X , Chaudhary A , Samaras P , Richter S , Shikata H & Messerer M (2020) Mass‐spectrometry‐based draft of the Arabidopsis proteome. Nature 579, 409–414. 32188942 10.1038/s41586-020-2094-2

[88] Papatheodorou I , Moreno P , Manning J , Fuentes AM‐P , George N , Fexova S , Fonseca NA , Füllgrabe A , Green M & Huang N (2020) Expression atlas update: from tissues to single cells. Nucleic Acids Res 48, D77–D83. 31665515 10.1093/nar/gkz947 PMC7145605

[89] Lu J , Wu T , Zhang B , Liu S , Song W , Qiao J & Ruan H (2021) Types of nuclear localization signals and mechanisms of protein import into the nucleus. Cell Commun Signal 19, 60. 34022911 10.1186/s12964-021-00741-y PMC8140498

[90] Bollman KM , Aukerman MJ , Park M‐Y , Hunter C , Berardini TZ & Poethig RS (2003) HASTY, the Arabidopsis ortholog of exportin 5/MSN5, regulates phase change and morphogenesis. Development 130, 1493. 12620976 10.1242/dev.00362

[91] Li J & Chen X (2003) PAUSED, a putative exportin‐t, acts pleiotropically in Arabidopsis development but is dispensable for viability. Plant Physiol 132, 1913–1924. 12913148 10.1104/pp.103.023291 PMC181277

[92] Luo Y , Wang Z , Ji H , Fang H , Wang S , Tian L & Li X (2013) An a rabidopsis homolog of importin β1 is required for ABA response and drought tolerance. Plant J 75, 377–389. 23582042 10.1111/tpj.12207

[93] Hodel AE , Harreman MT , Pulliam KF , Harben ME , Holmes JS , Hodel MR , Berland KM & Corbett AH (2006) Nuclear localization signal receptor affinity correlates with in vivo localization in *Saccharomyces cerevisiae* . J Biol Chem 281, 23545–23556. 16785238 10.1074/jbc.M601718200

[94] Hübner S , Smith HM , Hu W , Chan CK , Rihs H‐P , Paschal BM , Raikhel NV & Jans DA (1999) Plant importin α binds nuclear localization sequences with high affinity and can mediate nuclear import independent of importin β. J Biol Chem 274, 22610–22617. 10428841 10.1074/jbc.274.32.22610

[95] Kosugi S , Hasebe M , Entani T , Takayama S , Tomita M & Yanagawa H (2008) Design of peptide inhibitors for the importin α/β nuclear import pathway by activity‐based profiling. Chem Biol 15, 940–949. 18804031 10.1016/j.chembiol.2008.07.019

[96] Timney BL , Tetenbaum‐Novatt J , Agate DS , Williams R , Zhang W , Chait BT & Rout MP (2006) Simple kinetic relationships and nonspecific competition govern nuclear import rates in vivo. J Cell Biol 175, 579–593. 17116750 10.1083/jcb.200608141 PMC2064595

[97] Marfori M , Lonhienne TG , Forwood JK & Kobe B (2012) Structural basis of high‐affinity nuclear localization signal interactions with importin‐α. Traffic 13, 532–548. 22248489 10.1111/j.1600-0854.2012.01329.x

[98] de Oliveira HC , da Silva TD , Salvador GH , Moraes IR , Fukuda CA , de Barros AC & Fontes MR (2021) Structural and calorimetric studies reveal specific determinants for the binding of a high‐affinity NLS to mammalian importin‐alpha. Biochem J 478, 2715–2732. 34195786 10.1042/BCJ20210401

[99] Waese J , Fan J , Pasha A , Yu H , Fucile G , Shi R , Cumming M , Kelley LA , Sternberg MJ & Krishnakumar V (2017) ePlant: visualizing and exploring multiple levels of data for hypothesis generation in plant biology, the plant cell. Plant Cell 29, 1806–1821. 28808136 10.1105/tpc.17.00073 PMC5590499

[100] Rhee SY , Beavis W , Berardini TZ , Chen G , Dixon D , Doyle A , Garcia‐Hernandez M , Huala E , Lander G & Montoya M (2003) The Arabidopsis information resource (TAIR): a model organism database providing a centralized, curated gateway to Arabidopsis biology, research materials and community. Nucleic Acids Res 31, 224–228. 12519987 10.1093/nar/gkg076 PMC165523

[101] Ström A‐C & Weis K (2001) Importin‐beta‐like nuclear transport receptors. Genome Biol 2, 1–9. 10.1186/gb-2001-2-6-reviews3008 PMC138946 11423015

[102] Duncan RR (2006) Fluorescence lifetime imaging microscopy (FLIM) to quantify protein–protein interactions inside cells. Biochem Soc Trans 34, 679–682. 17052173 10.1042/BST0340679 PMC1855982

[103] Gonçalves Dias M , Dharmasena T , Gonzalez‐Ferrer C , Maika JE , Miguel VN , Dou R , Rodriguez Gallo MC , Bredow M , Siegel KR & Uhrig RG (2025) Catalytically inactive subgroup VIII receptor‐like cytoplasmic kinases regulate the immune‐triggered oxidative burst in *Arabidopsis thaliana* . J Exp Bot 76, 1553–1568. 39673241 10.1093/jxb/erae486 PMC11981898

[104] Maika JE , Krämer B , Strotmann VI , Wellmer F , Weidtkamp‐Peters S , Stahl Y & Simon R (2023) One pattern analysis (OPA) for the quantitative determination of protein interactions in plant cells. Plant Methods 19, 73. 37501124 10.1186/s13007-023-01049-3 PMC10375638

[105] Roy A , Kucukural A & Zhang Y (2010) I‐TASSER: a unified platform for automated protein structure and function prediction. Nat Protoc 5, 725–738. 20360767 10.1038/nprot.2010.5 PMC2849174

[106] Abramson J , Adler J , Dunger J , Evans R , Green T , Pritzel A , Ronneberger O , Willmore L , Ballard AJ & Bambrick J (2024) Accurate structure prediction of biomolecular interactions with AlphaFold 3. Nature 630, 493–500. 38718835 10.1038/s41586-024-07487-w PMC11168924

[107] Moreno P , Fexova S , George N , Manning JR , Miao Z , Mohammed S , Muñoz‐Pomer A , Fullgrabe A , Bi Y & Bush N (2022) Expression atlas update: gene and protein expression in multiple species. Nucleic Acids Res 50, D129–D140. 34850121 10.1093/nar/gkab1030 PMC8728300

