# Supplementary material for: The importin‐alpha superfamily engages in ethylene signaling by shuttling ETHYLENE INSENSITIVE 2 from the endoplasmic reticulum to the nucleus
Source: FEBS J. 2025 Oct 13;293(3):894–917. doi: 10.1111/febs.70285 (PMC12871931; doi:10.1111/febs.70285)
Supplement: Supplementary file 1 — Fig. S1. Sequence alignment of A. thaliana IMPα1‐9. Fig. S2. Alignment of amino acid sequences of the nine IMPα isoforms at the minor binding site. Fig. S3. Alphafold model of the IMPα1‐IMPβ1‐NOP‐1 complex. Fig. S4. IMPα protein family expression levels in A. thaliana after ACC treatment. Fig. S5. HPLC chromatogram of NOP‐1. Fig. S6. Mass spectrometry of NOP‐1. Fig. S7. HPLC chromatogram of N41P‐1. Fig. S8. Mass spectrometry of N41P‐1. Fig. S9. Reversed phase (RP) HPLC chromatogram of TAMRA‐NOP‐1. Fig. S10. Mass spectrometry analysis of TAMRA‐NOP‐1. Table S1. EIN2‐NLS peptide/protein sequences investigated in this study. Table S2. List of primers used for PCR amplification and cloning of IMPα genes. [file FEBS-293-894-s001.pdf]

Supporting Information

Linking ER to Nucleus in Ethylene Signaling: Role of the IMPα superfamily in nucleocytoplasmic transport of central regulator EIN2

Fabian Wynen, Jan Eric Maika, Raphael Josef Eberle, Nina Jahnke, Marcel Wiermer, Laura Hartmann, Rüdiger Simon and Georg Groth

Table of contents

Fig. S1. Sequence alignment of *A. thaliana* IMPα1-9.
Fig. S2. Alignment of amino acid sequences of the nine IMPα isoforms at the minor binding site using T-COFFEE.
Fig. S3. Alphafold model of the IMPα1-IMPβ1-NOP-1 complex.
Fig. S4. HPLC chromatogram of NOP-1.
Fig. S5. Mass spectrometry of NOP-1.
Fig. S6. HPLC chromatogram of N41P-1.
Fig. S7. Mass spectrometry of N41P-1.
Fig. S8. Reversed phase (RP) HPLC chromatogram of TAMRA-NOP-1.
Fig. S9. Mass spectrometry analysis of TAMRA-NOP-1.
Table S1. EIN2-NLS peptide/protein sequences investigated in this study.
Table S2. List of primers used for PCR amplification and cloning of IMPα genes.

```
IMPα1    MSLRPNAKTEVRRNRYKVAVDAEEGRRRREDNMVEIRKSKREESLMKKRREGMQALQ---    57
IMPα2    MSLRPNAKTEVRRNRYKVAVDAEEGRRRREDNMVEIRKSKREESLQKKRREGLQANQ-L-    58
IMPα4    MSLRPSTRAELRKKIYKTGVDADEARRRREDNLVEIRKNKREDSLLKKRREGMMLQQQL-    59
IMPα6    MSYKPSAKTEVRRNRYKVSVDADEGRRRREDNMVEIRKNKREENLQKKRREGFNPSMAS-    59
IMPα3    MSLRPSAKTEVRRNRYKVAVDAEEGRRRREDNLVEIRKNKREENLQKKRFTSSM-AFGS-    58
IMPα5    MSLRPSTKTEIRRIRYKVSVDAEEGRRRREDFLVEIRKSKRNENLMKKRRVKVLPPDYK-    59
IMPα7    --MKGGETMSVRRSGYKAVVDGVGGRRRREDDMVEIRKAKREESLLKKRREALPHSP---    55
IMPα8    ------------------------------------------------------------    0
IMPα9    --MADDGSASNRRDPIKSSVGNVAGQRRRKQAV-TVAKERRELLVRAKRLCRVGTNGDVE    57

IMPα1    --------------GFPS-ASAASVDKKLDSLKDMVAGVWSDDPALQLESTTQFRKLLSI    102
IMPα2    --------PQ--FAPSPV-PASSTVEKKLESLPAMVGGVWSDDRSLQLEATTQFRKLLSI    107
IMPα4    --------PL--GAGLDGPQTAAAVEKRLEGIPMMVQGVYSDDPQAQLEATTQFRKLLSI    109
IMPα6    --------QP--GQD---FSSSLPTETRLENIQQMIAGVMSEDRDLQLEATASFRRLLSI    106
IMPα3    --------AT--GQT---EQDLSSANQLKDNLPAMVAGIWSEDSNSQLEATNLLRKLLSI    105
IMPα5    --------LI-----------SNDPFESLLEIANMITGVFSDDPSLQLEYTTRFRVVLSF    100
IMPα7    --------------------SADSL------DQKLISCIWSDERDLLIEATTQIRTLLCG    89
IMPα8    ---------------------MAWKTEVNEVSDDIIDGLWSDDPPLQLESVTKIRRITSQ    39
IMPα9    DALVENEMMVDEEQPILEAQASKSV----EELKSAVQYQGKGAMQKRVTALRELRRLLSK    113

IMPα1    ER-SPPIEEVISAGVVPRFVEFLKKEDYPAIQFEAAWALTNIASGTSDHTKVVIDHNAVP    161
IMPα2    ER-SPPIEEVIDAGVVPRFVEFLTREDYPQLQFEAAWALTNIASGTSENTKVVIEHGAVP    166
IMPα4    ER-SPPIDEVIKAGVIPRFVEFLGRHDHPQLQFEAAWALTNVASGTSDHTRVVIEQGAVP    168
IMPα6    ER-NPPINEVVQSGVVPHIVQFLSRDDFTQLQFEAAWALTNIASGTSENTRVIIDSGAVP    165
IMPα3    EQ-NPPINEVVQSGVVPRVVKFLSRDDFPKLQFEAAWALTNIASGTSENTNVIIESGAVP    164
IMPα5    DR-SPPTDNVIKSGVVPRFVEFLKKDDNPKLQFEAAWALTNIASGASEHTKVVIDHGVVP    159
IMPα7    EMFNVRVEEVIQAGLVPRFVEFLTWDDSPQLQFEAAWALTNIASGTSENTEVVIDHGAVA    149
IMPα8    RD----ISCVIRSGVVPRLVQLLKNQVFPKLQYEVAWALTNIAV---DNPGVVVNNNAVP    92
IMPα9    SE-FPPVEAALRAGAIPLLVQCLSFGSPDEQLLESAWCLTNIAAGKPEETKALLP--ALP    170

IMPα1    IFVQLL-ASPSDDVREQAVWALGNVAGDSPRCRDLVLGCGALLPLLNQLNEHAKLSMLRN    220
IMPα2    IFVQLL-ASQSDDVREQAVWALGNVAGDSPRCRDLVLGQGALIPLLSQLNEHAKLSMLRN    225
IMPα4    IFVKLL-TSASDDVREQAVWALGNVAGDSPNCRNLVLNYGALEPLLAQLNENSKLSMLRN    227
IMPα6    LFVKLL-SSASEEVREQAVWALGNVAGDSPKCRDHVLSCEAMMSLLAQFHEHSKLSMLRN    224
IMPα3    IFIQLL-SSASEDVREQAVWALGNVAGDSPKCRDLVLSYGAMTPLLSQFNENTKLSMLRN    223
IMPα5    LFVQLL-ASPDDDVREQAIWGLGNVAGDSIQCRDFVLNSGAFIPLLHQLNNHATLSILRN    218
IMPα7    ILVRLL-NSPYDVVREQVVWALGNISGDSPRCRDIVLGHAALPSLLLQLNHGAKLSMLVN    208
IMPα8    VLIQLI-ASPKDYVREQAIWTLSNVAGHSIHYRDFVLNSGVLMPLLRLLYKDT---TLRI    148
IMPα9    LLIAHLGEKSSAPVAEQCAWAIGNVAGEGEDLRNVLLSQGALPPLARMIFP-DKGSTVRT    229

IMPα1    ATWTLSNFCRGKPQPHFDQ---VKPALPALERLIHSDDEEVLTDACWALSYLSDGTNDKI    277
IMPα2    ATWTLSNFCRGKPQPPFDQ---VRPALPALERLIHSTDEEVLTDACWALSYLSDGTNDKI    282
IMPα4    ATWTLSNFCRGKPPTPFEQ---VKPALPILRQLIYLNDEEVLTDACWALSYLSDGPNDKI    284
IMPα6    ATWTLSNFCRGKPQPAFEQ---TKAALPALERLLHSTDEEVLTDASWALSYLSDGTNEKI    281
IMPα3    ATWTLSNFCRGKPPPAFEQ---TQPALPVLERLVQSMDEEVLTDACWALSYLSDNSNDKI    280
IMPα5    ATWTLSNFFRGKPSPPFDL---VKHVLPVLKRLVYSDDEQVLIDACWALSNLSDASNENI    275
IMPα7    AAWTLSNLCRGKPQPPFDQ---VSAALPALAQLIRLDDKELLAYTCWALVYLSDGSNEKI    265
IMPα8    ATWALRNLCRGKPHPAFDQ---VKPALPALEILLHSHDEDVLKNACMALCHLSEGSEDGI    205
IMPα9    AAWALSNLIKGPESKAAAQLVKIDGILDAILRHLKKTDEETATEIAWIIVYLSALSDIAT    289
```

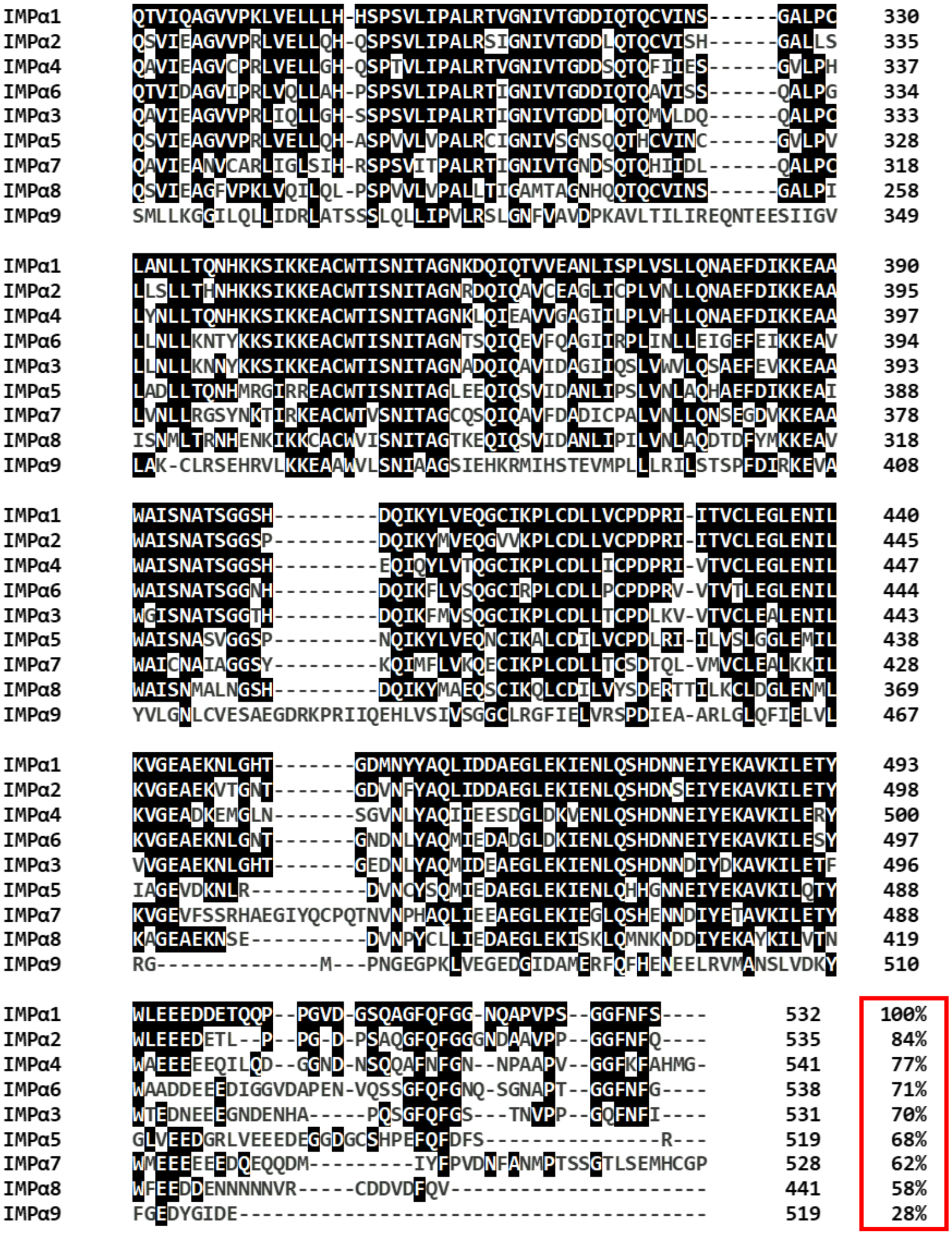

Fig. S1. Sequence alignment of *A. thaliana* IMPα1-9. Sequence identity to IMPα1 is shown in %. IMPα2-8 possess a sequence identity to IMPα1 >50%, in contrast the sequence identity of IMPα9 is <30%. IMPα1 (UniProt: Q96321), IMPα2 (UniProt: F4JL11), IMPα3 (UniProt: O04294), IMPα4 (UniProt: O80480), IMPα5 (UniProt: Q9FJ09), IMPα6 (UniProt: Q9FWY7), IMPα7 (UniProt: Q9M9X7), IMPα8 (UniProt: Q9FJ92), IMPα9 (UniProt: F4KF65). Alignments were made using the MUSCLE tool from EMBL-EBI.

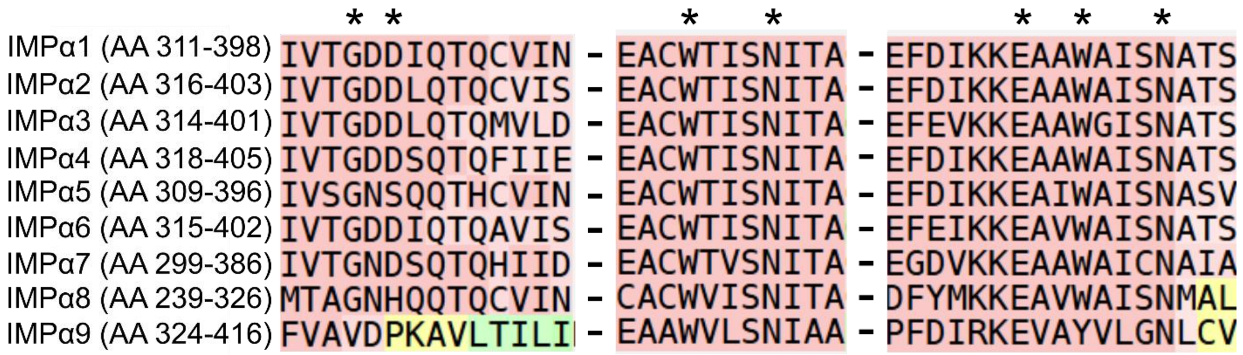

Fig. S2. Alignment of amino acid sequences of the nine IMPα isoforms at the minor binding site using T-COFFEE. Potential residues involved in NOP-1 binding are marked with asterisks (Related to Fig. 3). Pink yellow IMPα1 (UniProt: Q96321), IMPα2 (UniProt: F4JL11), IMPα3 (UniProt: O04294), IMPα4 (UniProt: O80480), IMPα5 (UniProt: Q9FJ09), IMPα6 (UniProt: Q9FWY7), IMPα7 (UniProt: Q9M9X7), IMPα8 (UniProt: Q9FJ92), IMPα9 (UniProt: F4KF65). Alignments were made using the MUSCLE tool from EMBL-EBI.

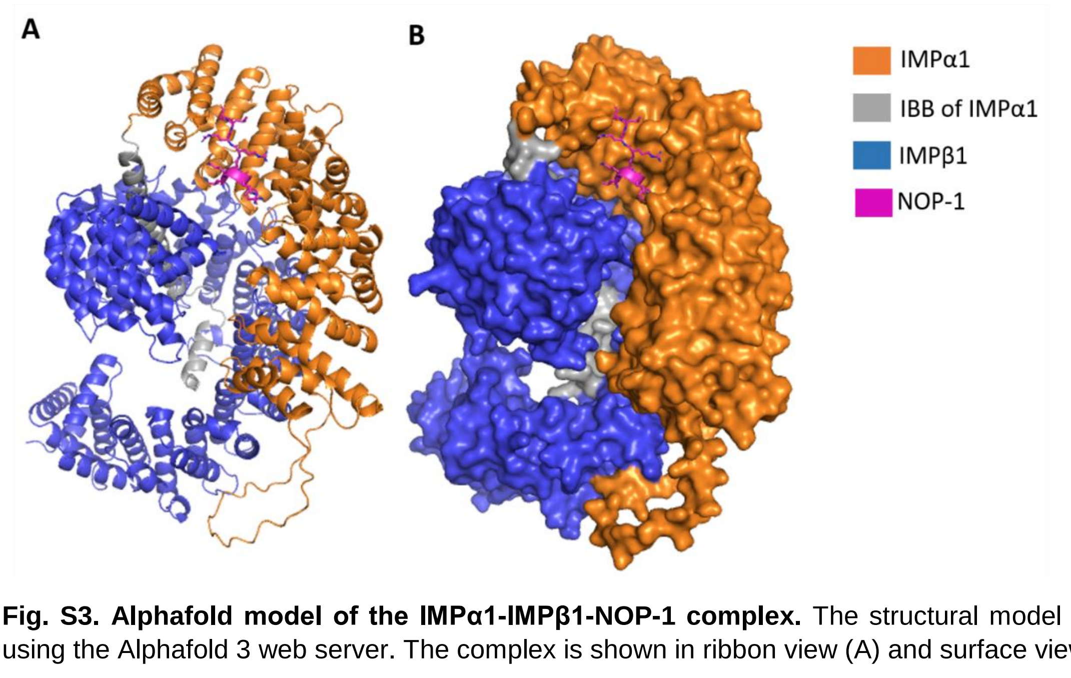

Fig. S3. Alphafold model of the IMPα1-IMPβ1-NOP-1 complex. The structural model was generated using the Alphafold 3 web server. The complex is shown in ribbon view (A) and surface view (B).

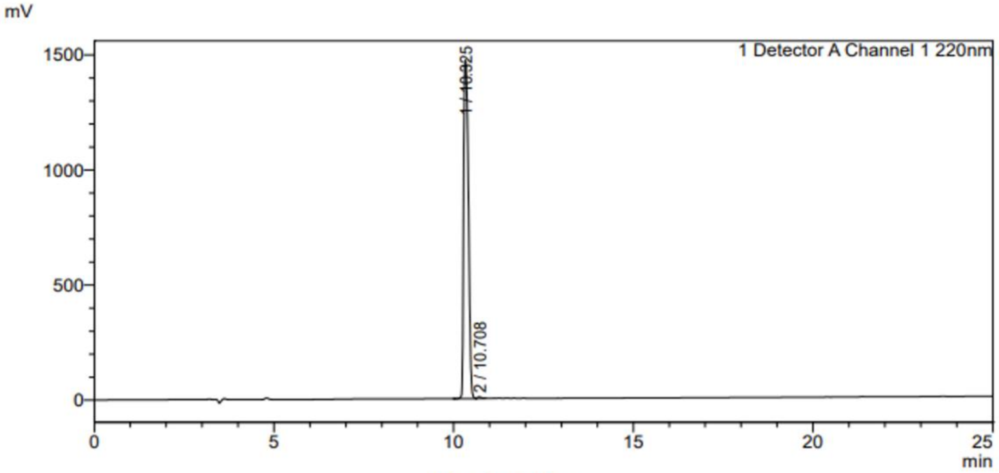

<Peak Table>

Detector A Channel 1 220nm

Peak#	Ret. Time	Area	Height	Area%
1	10.325	13660621	1470725	99.595
2	10.708	55578	7105	0.405
Total		13716199	1477829	100.000

Fig. S4. HPLC chromatogram of NOP-1. Information obtained from the purity and quality certificate from GenScript (New Jersey, USA).

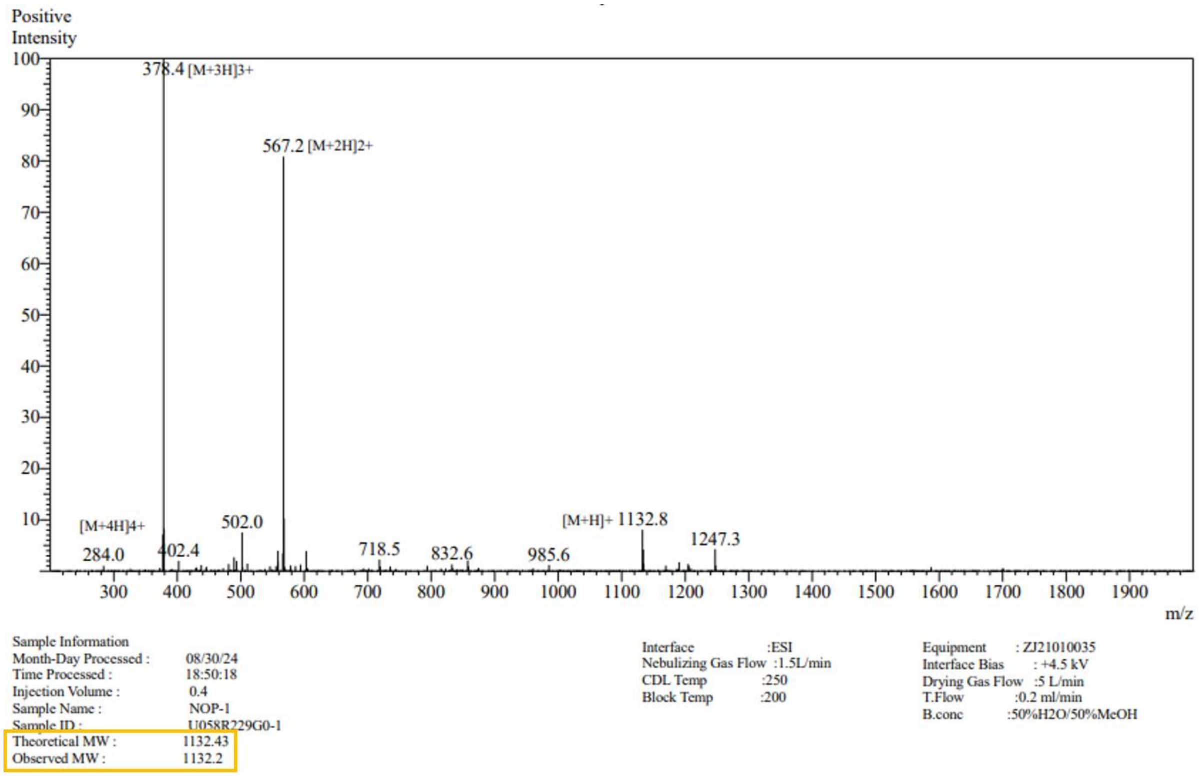

Fig. S5. Mass spectrometry of NOP-1. Information obtained from the purity and quality certificate from GenScript (New Jersey, USA).

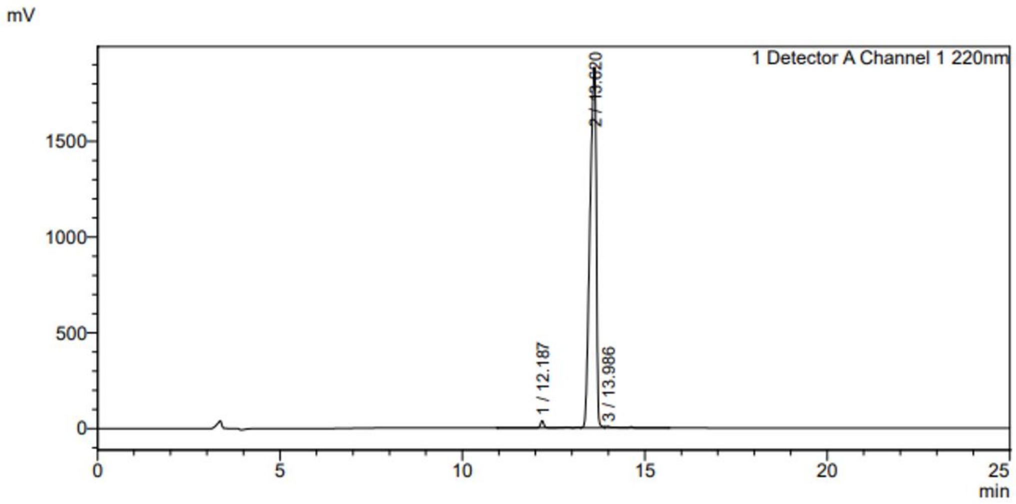

<Peak Table>

Detector A Channel 1 220nm

Peak#	Ret. Time	Area	Height	Area%
1	12.187	313557	37124	1.353
2	13.620	22694476	1884833	97.921
3	13.986	168213	7556	0.726
Total		23176246	1929512	100.000

Fig. S6. HPLC chromatogram of N41P-1. Information obtained from the purity and quality certificate from GenScript (New Jersey, USA).

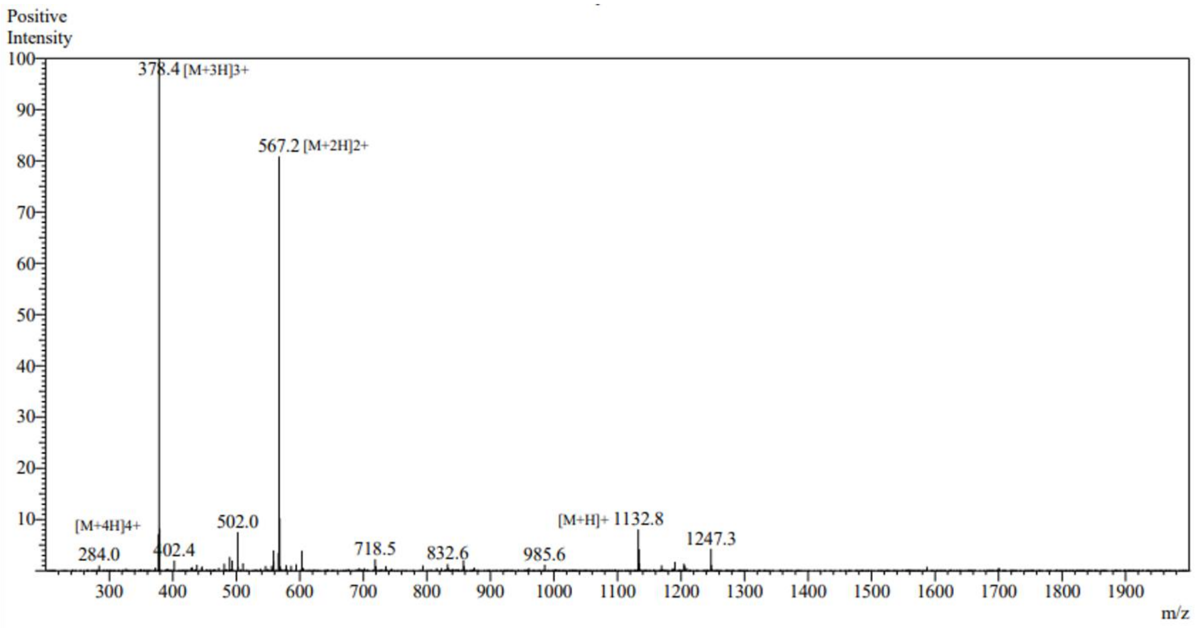

Sample Information
Month-Day Processed : 08/30/24
Time Processed : 18:50:18
Injection Volume : 0.4
Sample Name : NOP-1
Sample ID : U058R229G0-1
Theoretical MW : 1132.43
Observed MW : 1132.2

Interface :ESI
Nebulizing Gas Flow :1.5L/min
CDL Temp :250
Block Temp :200

Equipment : ZJ21010035
Interface Bias : +4.5 kV
Drying Gas Flow :5 L/min
T.Flow :0.2 ml/min
B.conc :50%H2O/50%MeOH

Fig. S7. Mass spectrometry of N41P-1. Information obtained from the purity and quality certificate from GenScript (New Jersey, USA).

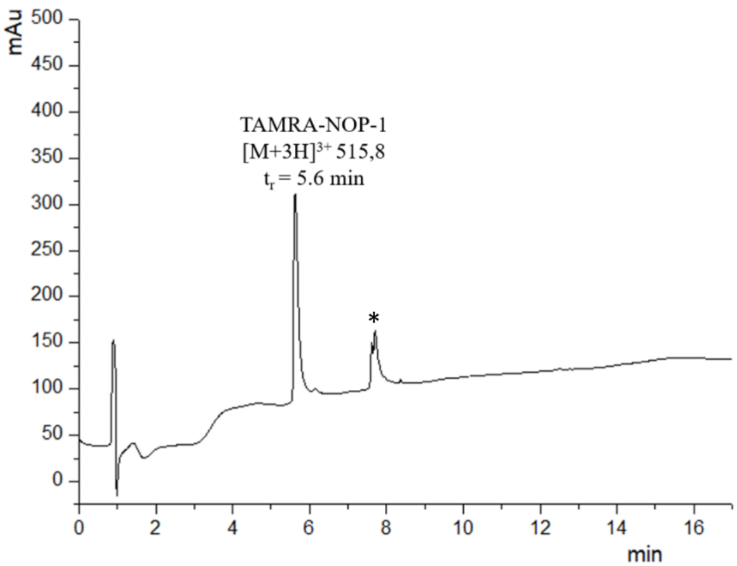

Fig. S8. Reversed phase (RP) HPLC chromatogram of TAMRA-NOP-1. *Byproduct in which the arginine Pbf protecting group was not fully removed during deprotection (TAMRA-NOP-1 +Pbf).

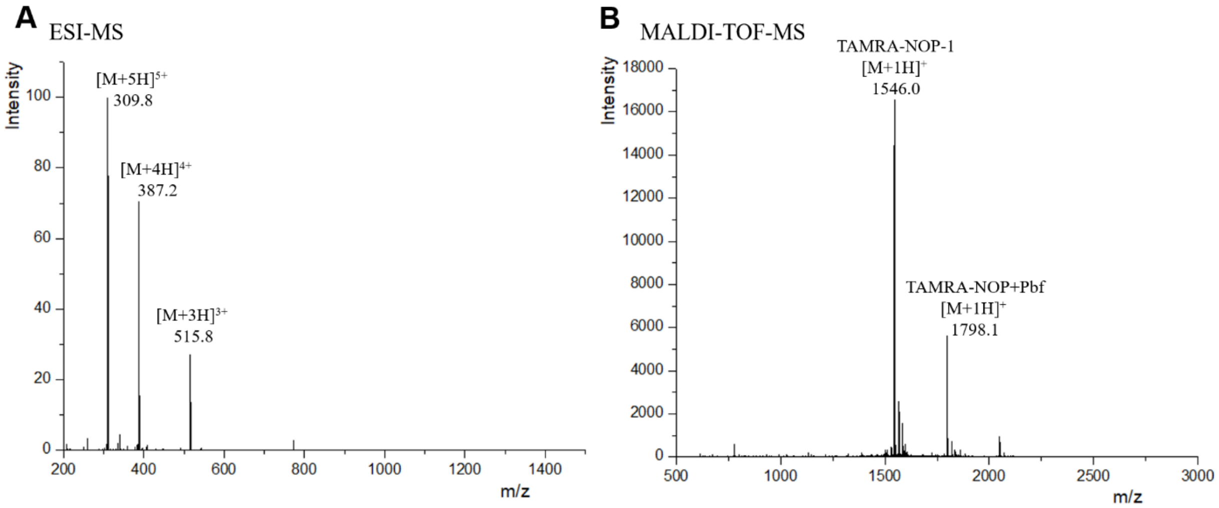

Fig. S9. Mass spectrometry analysis of TAMRA-NOP-1. (A) ESI-MS and (B) MALDI-TOF-MS.

Table S1. EIN2-NLS peptide/protein sequences investigated in this study. The NLS is highlighted in red, while basic amino acids within the NLS are marked in bold. The GB1-domain is highlighted in blue.

Name	Sequence
NOP-1	L**KR**Y**KRR**L
TAMRA-NOP-1	TAMRA-L**KR**Y**KRR**L
N41P-1	KDVEMAISSRKGRTGTAAGDVAFPKGKENLASVL**KR**Y**KRR**L
GB1-*At*EIN2C_1215-1281	MQYKLILNGKTLKGETTTEAVDAATAEKVFKQYANDNGVDGEWTYDDA TKTYTVTESGSENLYFQGHKGKCTTAVTLLDLIKDVEMAISCRKGRTGT AAGDVAFPKGKENLASVL**KR**Y**KRR**LSNKPVGMNQDGP

Table S2. List of primers used for PCR amplification and cloning of *IMPα* genes. Genes were either cloned into pETEV21a (*IMPα1-4*/*6-9*) or pETEV16b (*IMPα5*).

Primername	Primersequence (5'->3')
IMPα1_for	ctttaagaaggagatatacaATGTCACTGAGACCCAAC
IMPα1_rev	caggttttcggatcccatatgGCTGAAGTTGAATCCTCC
ΔIBB_IMPα1_for	ctttaagaaggagatatacaATGCTGAAGGATATGGTCGC
ΔIBB_IMPα1_rev	caggttttcggatcccatatgGCTGAAGTTGAATCCTCC
IMPα2_for	ctttaagaaggagatatacaATGTCTTTGAGACCTAAC
IMPα2_rev	caggttttcggatcccatatgCTGGAAGTTGAATCCACC
IMPα3_for	ctttaagaaggagatatacaATGTCTCTCAGACCTAGC
IMPα3_rev	caggttttcggatcccatatgAATAAAGTTGAATTGACC
IMPα4_for	ctttaagaaggagatatacaATGTCGCTGAGGCCGAGC
IMPα4_rev	caggttttcggatcccatatgGGCAAATTTGAATCCACC
IMPα5_for	ctttaagaaggagatatacaATGTCCTTGCGACCGAGC
IMPα5_rev	caggttttcggatcccatatgACGAGAAAAATCAAACTGGAATTCCG
IMPα6_for	ctttaagaaggagatatacaATGTCTTACAAACCAAGCG
IMPα6_rev	caggttttcggatcccatatgACCAAAGTTGAATCCAC
ΔIBB_IMPα7_for	ctttaagaaggagatatacaATGAGGGATCTGTTGATTG
ΔIBB_IMPα7_rev	caggttttcggatcccatatgAGGTCCGCAGTGCATCTC
IMPα8_for	ctttaagaaggagatatacaATGGCTTGGAAAACAGAG
IMPα8_rev	caggttttcggatcccatatgCACCTGAAAGTCCACATC
IMPα9_for	ctttaagaaggagatatacaATGGCGGATGATGGCTC
IMPα9_rev	caggttttcggatcccatatgTTCATCGATTCCATAATC
